# Controlled image-based prediction of soil nutrient concentrations (NPK) using machine and deep learning

**DOI:** 10.1371/journal.pone.0360517

**Published:** 2026-10-27

**Authors:** Umair Maqsood, Tehmina Karamat Ullah Khan, NZ Jhanjhi, Kashif Sattar, Fatima Iftikhar, Najma Imtiaz Ali, Raja Majid Mehmood

**Affiliations:** 1 School of Computer Science, Taylors University, Subang Jaya, Malaysia; 2 Foundation University, Islamabad, Pakistan; 3 School of Computing and Artificial Intelligence, Faculty of Engineering and Technology, Sunway University, Bandar Sunway, Selangor Darul Ehsan, Malaysia; 4 Office of Research and Development, Asia University, Taichung, Taiwan; 5 University Institute of Information Technology, PMAS Arid Agriculture University, Rawalpindi, Pakistan; 6 Department of Engineering Technology, Foundation University, Islamabad, Islamabad, Pakistan; 7 Department of Applied Data Engineering, Fakulti Teknologi Maklumat dan Komunikasi, Universiti Teknikal Malaysia, Melaka, Malaysia; 8 Centre for Advanced Analytics, COE for Artificial Intelligence, Multimedia University, Persiaran Multimedia, Cyberjaya, Selangor, Malaysia; 9 Faculty of Computing and Informatics, Multimedia University, Persiaran Multimedia, Cyberjaya, Selangor, Malaysia; University of Minnesota, UNITED STATES OF AMERICA

## Abstract

Soil nutrient analysis is essential for sustainable agriculture and precision farming. Traditional chemical tests for nitrogen, phosphorus, and potassium are reliable but costly, time-consuming, and often unavailable in remote areas. The objective of this study is to develop a novel approach that combines laboratory chemical extraction, controlled image acquisition, image processing, machine learning, and deep learning to classify soil nitrogen, phosphorus, and potassium levels. 50 independent soil samples were analyzed, chemically extracted, and classified as Low, Medium, or High for each nutrient. A specially designed black-box prototype was developed to capture high-quality images of soil extract samples under consistent lighting conditions. The images were used to extract color and texture information, which was evaluated using multiple classical machine-learning algorithms, such as K-nearest neighbours, support vector machines, logistic regression, random forest, extra trees, gradient boosting, and Gaussian naive Bayes. A convolutional neural network-based model was also used for comparison. To minimize data leakage, all validation procedures were performed at the physical-sample level, and augmented images were restricted to the training data. The best models obtained using nested cross-validation for nitrogen, phosphorus, and potassium were KNN, Random Forest, and Logistic Regression, respectively, with accuracies of 60%, 82%, and 48%. The nitrogen model correctly identified the High category, and the phosphorus model correctly identified the dominant Low category each time. For potassium, Logistic Regression made predictions of all three classes and outperformed the majority class baseline. The Classical machine-learning models slightly outperformed the compact CNN for nitrogen and potassium, while both achieved the same accuracy for phosphorus; however, overall performance remained limited by the small and imbalanced dataset. The findings suggest that the proposed framework could contribute to image-assisted decision-making in smart farming and support improved soil management practices, particularly in regions where laboratory testing facilities are limited.

## Introduction

Agriculture is a key supporter of the economy in countries where a large portion of the population depends on it for their livelihood [1]. It greatly impacts the social structure and environmental stability of these countries. As long as human societies develop, agriculture remains a key factor in supporting economic growth and sustainability [2]. Recent studies highlight that smart farming techniques driven by IoT and AI are becoming essential for sustaining agricultural productivity [3]. This domain is wide and complex, and many techniques are available to address this challenge in real-time. Assessing soil quality, monitoring crop health, managing periodic agricultural activities, and managing related activities are extremely important aspects of the agriculture sector [4]. Worldwide, agriculture accounts for 70% of water consumption, which is alarmingly dangerous for the potable water resources of more than 2.1 billion individuals [5,6]. Evolution in agricultural research validates that IoT and AI-based smart sensing technologies are transforming monitoring and decision-making practices [7]. Recent research further indicates that IoT-enabled agronomic systems provide real-time field intelligence, supporting precision interventions such as autonomous operations and nutrient management [8]. Soil is regarded as a fundamental resource supporting life on Earth [9]. Technological advancements, particularly in sensing and data-driven methods, have resulted in significant progress in agriculture, and soil also plays an essential role in agriculture, providing all the necessary conditions required for plant establishment, healthy root development, and successful seed germination [10,11]. According to FAOSTAT, more than 95% of global food depends directly or indirectly on soil [12]. Recent models emphasize soil fertility estimation using ensemble machine learning approaches, confirming soil’s critical role in ensuring food security [13]. It is crucial to identify soil samples accurately, as this is necessary for the proper supervision and rational use of arable lands, since soil samples possess varying physical and environmental structures and characteristics [14,15].

The three nutrients, nitrogen (N), phosphorus (P), and potassium (K), collectively called NPK, are fundamental to improving crop productivity [13]. Insufficient understanding of soil nutrient measures can lead to excessive application of fertilizers by farmers, which can result in soil fertility degradation due to the depletion or improvement of nutrient content [16]. The fertility of soil is a key factor in promoting healthy crops and maintaining agricultural productivity, and commonly evaluating soil quality has involved laboratory testing for essential nutrients such as N, P, and K. Even though accurate, these approaches are usually time-consuming, expensive [17], and not easily accessible to small-scale farmers [18], and soil analysis needs to be carried out by trained professionals [19]. Traditional testing relies on specialized equipment and chemical procedures, which increases both the cost and the risks associated with handling harmful substances [20]. To find the NPK content in the soil, sending soil samples for laboratory testing is the most common approach. However, this method is too hectic, and the accuracy of the results may be affected by changes in soil conditions during transportation from the farm to the laboratory. Moreover, most available test kits require technical expertise to use effectively. Therefore, the development of an accessible and practical soil analysis system is necessary to support crop production [21].

Laboratory soil testing mainly involves various steps, such as collecting soil samples from different locations, mixing them with appropriate amounts of chemicals and reagents, analyzing the nutrient content of NPK in the given sample, and providing recommendations based on results. It is important to note that the level of nutrients in soil can vary depending on soil, weather conditions, and the type of crop being cultivated [22]. This study focuses on three primary nutrients found in soil, namely NPK. These are very important for plant growth, with N promoting leaf and vegetation growth, P promoting overall plant and root growth, and K regulating nutrients and water in plant cells and promoting flowering and fruiting. Although some low-cost sensors can measure a certain mineral content in the soil, commercial chemical tests are necessary to accurately measure NPK content. Soil extracts for nutrient analysis were prepared for each soil type, images of these extracts were captured under uniform lighting conditions, and separate models were trained for each soil category. The trained models were evaluated with independent sample-level validation to classify the N, P, and K status as Low, Medium, and High. This study is based on the soil extract image captured under controlled lighting conditions for the classification of the NPK status, compared to studies that analyze the raw soil images. The proposed approach establishes a controlled laboratory framework for image-based NPK classification and provides a foundation for broader validation across additional soil types and geographic regions. The key findings of this study are as follows:

- **Controlled Soil-Extract Imaging Framework:** Developed a standardized black-box imaging setup for capturing visible color information from chemically prepared soil extracts under controlled lighting conditions.
- **Laboratory-Referenced NPK Classification:** Conducted conventional chemical soil analysis to categorize the soils as Low, Medium, and High reference classes for nitrogen, phosphorus, and potassium (NPK).
- **Comparative ML and CNN Evaluation:** Evaluated classical machine learning (ML) models and a convolutional neural network (CNN) to examine the suitability of soil extract images for NPK status classification.
- **Leakage-Controlled Sample-Level Validation:** Distinguished the 50 independent physical soil samples from their enhanced image forms and limited augmentation to training data to prevent leakage.
- **Imbalance-Aware Performance Assessment:** The models were evaluated with accuracy, balanced accuracy, macro-F1 score, Cohen’s kappa, Matthews Correlation Coefficient (MCC), confidence intervals, majority-class baselines, and confusion matrices.

### Related work

This section provides an overview of existing techniques developed for soil nutrient analysis, focused on Nitrogen, phosphorus, and Potassium (NPK). In [23], the simultaneous measurement of NPK ions in soil water extracts was achieved using a potentiometric multisensor array. The authors performed multivariate data analysis techniques in order to process sensor responses. Also, correlation coefficients ranging from 0.69 to 0.96 were calculated between the targeted parameters and sensor responses. This study demonstrated that the nitrogen concentration could be accurately determined within a range of 60−426 mg/kg, with a root mean square error of 50 *mg*/*kg*. Another study [24] utilized color sensors paired with a NodeMCU microcontroller in order to quantify the levels of NPK in soil samples. They developed a system named the “Smart Soil Quality Predictor”. Smart Soil Quality Predictor was designed to detect and quantify essential macronutrients in the soil. The sensor setup included a photodiode for light detection and four LEDs to ensure consistent illumination during image acquisition. Whereas conventional NPK test kits rely on liquid extracts, the authors adapted their method for dry soil samples, reducing cost and simplifying field deployment. A statistical approach was used by applying multiple Linear Regression (MLR) to determine soil macronutrients. Using MLR, the authors [16] established a relationship between primary and secondary soil parameters, that is, pH, electrical conductivity, and NPK. This model enabled the estimation and correlation of NPK values. It achieved an accuracy of roughly 80%.

ML & DL related methods for recommending strategic crops and predicting soil nutrients were presented in [25]. They discussed problems related to soil degradation caused by hard farming methods and suggested models based on data to make agriculture more sustainable. The study revealed that CNN and LSTM deep learning models performed better than traditional models in predicting nutrient parameters such as pest occurrence, fertilizer use, soil health, and climatic changes with an accuracy of more than 90%. In [26], researchers aimed to explore the feasibility of classifying soil nutrient content using soil images as compared to traditional laboratory testing techniques. They used the Gray Level Co-Occurrence Matrix (GLCM) to identify features describing the soil texture, and classified the nutrient content using the K-Nearest Neighbours (KNN) algorithm. Validation was performed by comparing it with laboratory nutrient testing data. The model was trained using 693 soil images and tested using 297 soil images. The results indicated 90.57% accuracy for nitrogen, 92.93% for phosphorus, and 91.92% for potassium.

The authors [27] applied five machine learning methods to the classification of appropriate crops and nutrients. Soil characteristics such as NPK, soil pH, and climatic factors such as temperature, rainfall, and humidity were also included in the dataset. The classifiers used for the evaluation were support vector machine, extreme gradient boosting, k-nearest neighbors, random forest, and decision tree classifiers. The study concluded that the accuracy of the models is higher when the agricultural and horticultural data were analyzed independently. Among the models compared, XGBoost had the best performance. Soil texture and nine key nutrients (clay, silt, Ca, Mg, C, P, N, ${\text{NO}}_{3}^{-}$ and NH_4_) were analyzed with open-source remote sensing imagery combined with ground truth data [28]. The dark object subtraction 1 (DOS1) method was used to reduce the level of noise from the atmosphere. They used four machine learning ensemble models: AdaBoost (ADA), Random Forest (RFR), Gradient Boosting (GB), and Extreme Gradient Boosting (XGB), and these models were evaluated to determine soil properties and to compare the accuracy of predictions for preprocessed and raw multispectral images.

In this study [29], two methods were presented to predict the NPK content in the soil, which is important for the quality and yield of crops. The first technique used pH sensors and electrical conductivity to measure soil parameters, and machine learning approaches were applied to find NPK values. The second technique used optical sensors to measure the quantity of light absorbed and reflected by the soil, and regression algorithms were used to calculate NPK. The NPK values were classified into different classes, and in both cases, an accuracy greater than 75% was achieved. An IoT-embedded system for soil nutrient analysis and crop recommendation to support smart farming decisions was developed [30]. A low-cost sensor setup collected data on soil moisture, temperature, pH, and NPK levels using color sensors and soil testing tablets. This data was stored in Firebase and accessed via a mobile app for continuous monitoring. A classification model using a multi-class SVM with a directed acyclic graph was enhanced using the fruit fly optimization algorithm. The high classification accuracy of 97.3% was achieved, while outperforming traditional SVM and decision tree models. A novel stacked ensemble learning architecture for the accurate estimation of soil fertility levels based on the macronutrients NPK was developed [13]. They added an improved model structure for a preprocessing stage, along with a feedback mechanism that lets the data inform the system as it runs. The model was validated using real field datasets and achieved higher performance compared to existing methods, with an average accuracy of 84.57% score of 0.6114 on Cohen’s kappa scale.

Several existing studies have shown high prediction accuracy, but they were performed with varying input types such as raw soil images, sensor measurements, remote sensing data, and structured soil variables. The differences in these studies also included class distribution, sample size, pre-processing, as well as validation. Therefore, they were not directly comparable with the present controlled soil-extract imaging study in terms of reported accuracy. To differentiate the proposed study from existing approaches, a comparative summary of related work is provided in Table 1. It highlights the methodology, use of image processing, machine learning, deep learning techniques, and novelty of each referenced study. The present study differs from previous studies in that it analyzes chemically prepared soil extract images under well-controlled lighting conditions, and it evaluates both machine-learning and deep-learning models for classification of soil extract images in terms of NPK status.

**Table 1:** Comparison of existing NPK prediction techniques and the proposed study.

Ref.	Study approach	Image processing	Models used	Performance	Extract imaging	Contribution
[23]	Potentiometric sensors	No	Multivariate analysis	RMSE ~50 mg/kg	No	Rapid sensor-fusion analysis
[24]	TCS3200 sensor with NodeMCU	No	–	Not reported	No	Sensor-based nutrient assessment
[16]	MLR with soil parameters	No	MLR	~80%	No	Regression using laboratory values
[25]	Structured soil and sensor data	No	CNN, LSTM	> 90%	No	Deep learning on sensor data
[26]	GLCM features from raw soil images	Yes	kNN	N: 90.5%; P: 92.9%; K: 91.9%	No	Classification from raw soil images
[27]	Crop and nutrient recommendation	No	XGBoost, SVM, RF, KNN, DT	AUC up to 1.0	No	Soil and climate datasets
[28]	Remote-sensing imagery	Indirect	RF, GB, XGB	Moderate	No	Satellite-based estimation
[29]	Optical and EC sensors	No	Neural networks	> 75%	No	Indirect EC/pH estimation
[30]	IoT and colour sensors	Limited	SVM, DAG, optimisation	97.3%	No	Mobile application integration
[13]	Stacked prediction framework	No	Ensemble ML	High	No	Advanced model stacking
**Ours**	**Controlled soil-extract image-based NPK classification**	**Yes**	**KNN, SVM-RBF, LR, RF, Extra Trees, GB, Gaussian NB**	**N: 60%; P: 82%; K: 48%**	**Yes**	**Controlled soil-extract imaging under standardised lighting**

## Materials and methods

This study used a dual-method technique to evaluate the concentrations of crucial soil-based nutrients such as NPK. The first method utilized conventional chemical analysis, while the second method adopted digital image processing, Machine Learning & Deep Learning to predict nutrient levels depending on their color features, which are extracted using soil extract images. Overall, the workflow is presented in Fig 1, providing a visual summary of the major stages.

**Fig 1:**
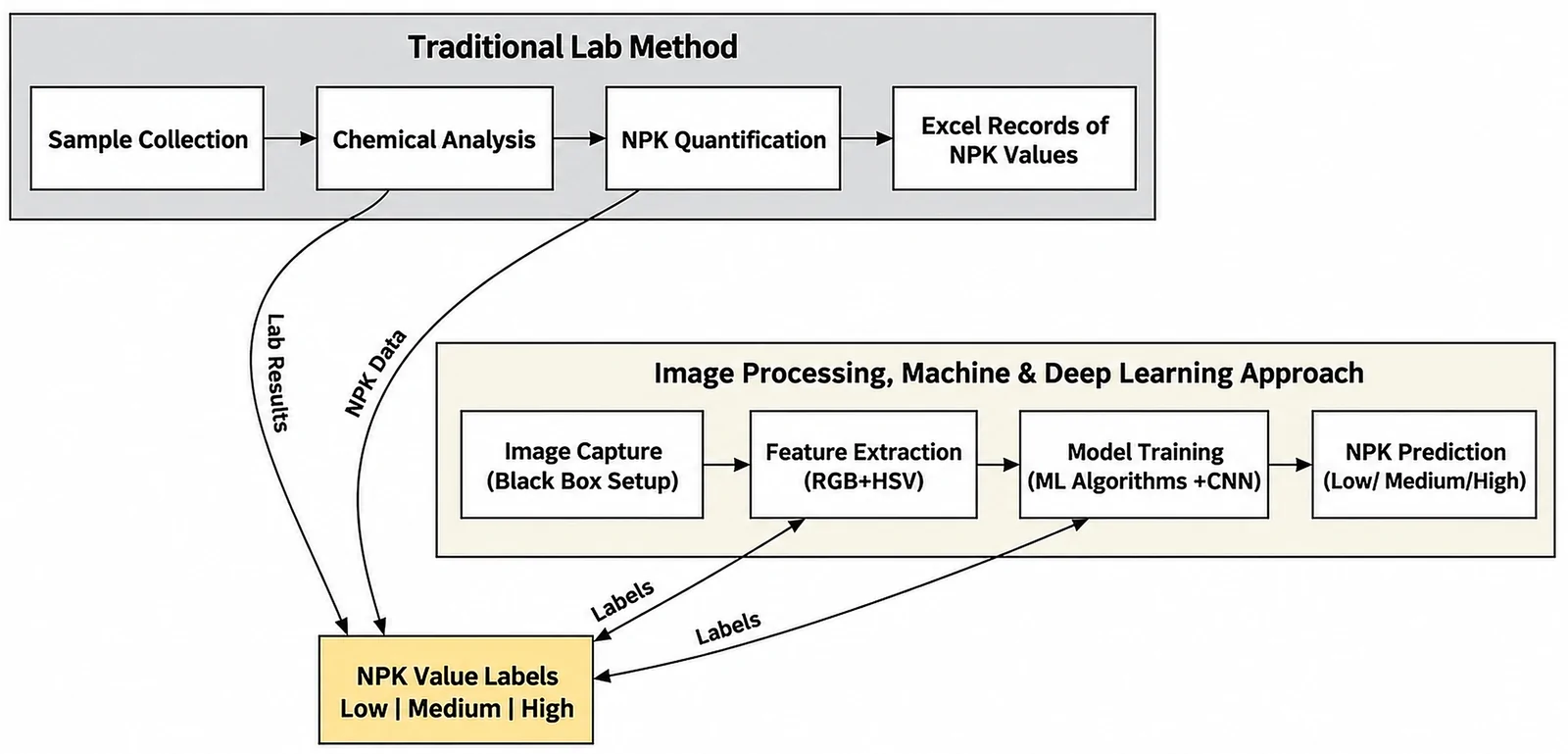
Workflow illustrating the step-by-step methodology for soil nutrient analysis.

### Overall research design

The study was conducted in the laboratory using 50 independent physical soil samples. Image augmentation was only applied as a training procedure and was not considered an increase in the number of independent observations. In order to determine the nitrogen, phosphorus, and potassium status as Low, Medium, or High, each model was evaluated individually.

The overall research is divided into two stages: The first stage is traditional laboratory analysis, and the second is image based classification. Based on the laboratory measurements, Low, Medium, and High nutrient status classes were assigned.

1. **Traditional Laboratory Method:** In this Soil nutrient concentrations were determined through standard chemical extraction procedures. A Kjeldahl apparatus was used for Nitrogen, a spectrophotometer was used for Phosphorus, and a flame photometer was used for Potassium. Then, based on the measured concentrations, each nutrient was classified into Low, Medium, or High categories.
2. **Image Based Classification:** To ensure consistency across samples, a black box imaging setup was built to photograph the chemically prepared soil extracts under controlled lighting. From these images, RGB and HSV color features were extracted, which then served as the input for training and evaluating several supervised ML and DL models independently for NPK status classification.

### Soil sampling

Soil samples were collected from multiple agricultural regions in Punjab at two depths: 0–15 cm and 15–30 cm. The debris and stones were removed, and the samples were mixed well to ensure homogeneity. It is essential to remove any debris or stones from soil samples, as they can affect the accuracy of the study and can cause damage to equipment during subsequent processing steps.

### Field-site access and permits

Soil samples were taken on privately operated agricultural land, with the consent of landowners or field managers. Sampling focused only on soil samples, and protected areas, endangered species, human subjects, and culturally sensitive sites were not included. For this reason, no formal government/institutional field-collection authorization was required. Sampling activities were completed with permission of the appropriate site personnel and with no harm to crops and agricultural infrastructure.

### Sample preparation

Upon collection, soil samples were dehydrated at room temperature to remove excess moisture. The debris and stones were removed, and the samples were mixed well to ensure homogeneity. It is essential to remove any debris or stones from soil samples, as they can affect the accuracy of the study and can cause damage to equipment during subsequent processing steps.

### Grinding and sieving

For Soil Nutrient analysis, dried and prepared soil samples were ground using an electric grinder until they were fine enough to pass through a 2 mm (about 0.08 in) sieve. These sieved samples were stored in cool, clean, and dry containers.

### Oven drying

After collection, the Soil Samples were dried in an oven at 105 °C for 24 hours to ensure thorough removal of any remaining moisture. Oven drying is very important for obtaining accurate measurements of soil properties. Any remaining moisture could potentially impact the results.

### Experimental setup

This section describes the procedures, tools, and environment used to carry out the study. The setup is designed to ensure reliable results for soil nutrient classification. Soil samples were collected and analyzed in the laboratory following standard chemical methods. The steps involved in preparing the soil extracts and carrying out the subsequent testing are outlined below.

### Soil extract preparation

To obtain the soil extract, 50 soil samples were taken and prepared for the extraction process. Each sample weighed approximately 10 grams, as shown in Fig 2.

**Fig 2:**
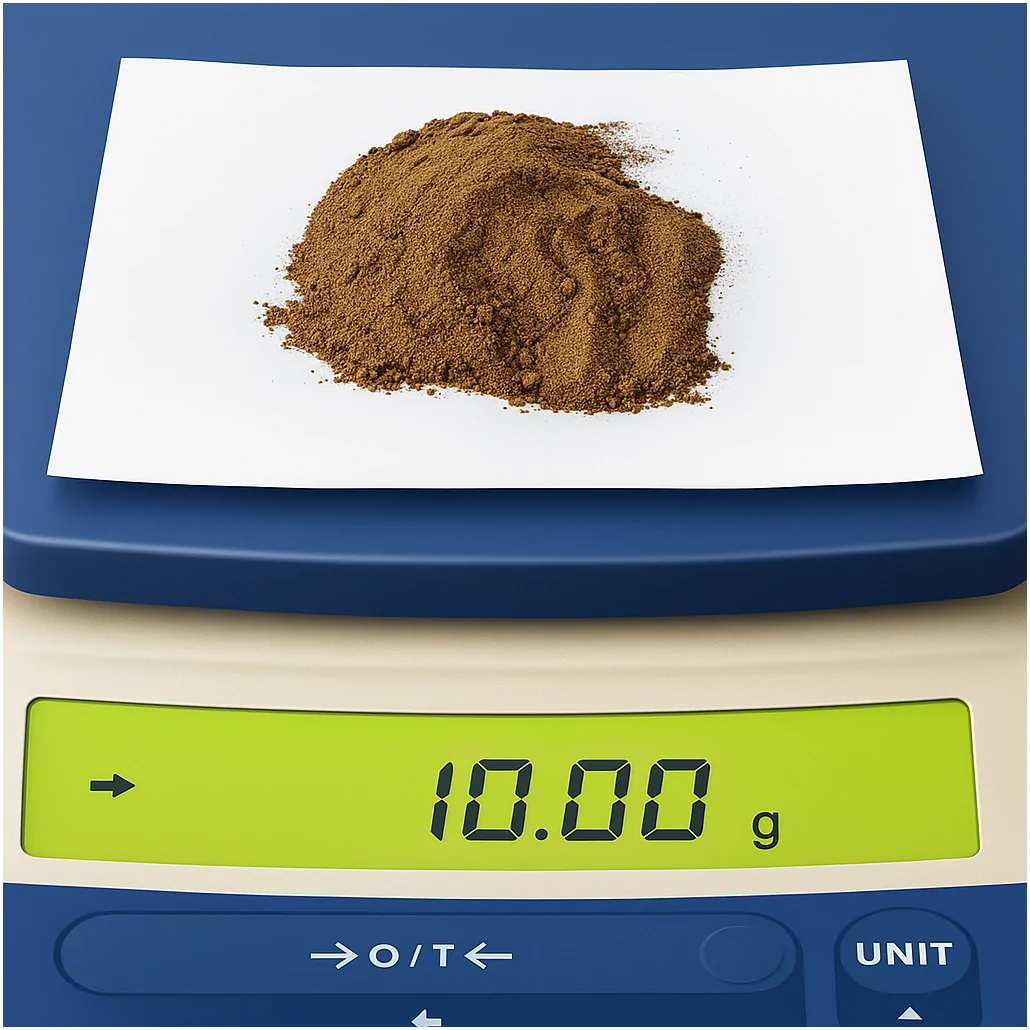
Soil weight measurement.

In order to prepare the AB-DTPA solution. 20 g of Diethylene triaminepentaacetic (DTPA) was dissolved in 1 liter of distilled water. Then, 2 g each of sodium bicarbonate and potassium chloride, and 0.5 g of calcium chloride were added and mixed thoroughly. The pH value was adjusted from 7.6 to 7.0 before mixing with soil, using NaOH for the given solution. It is shown in Fig 3.

**Fig 3:**
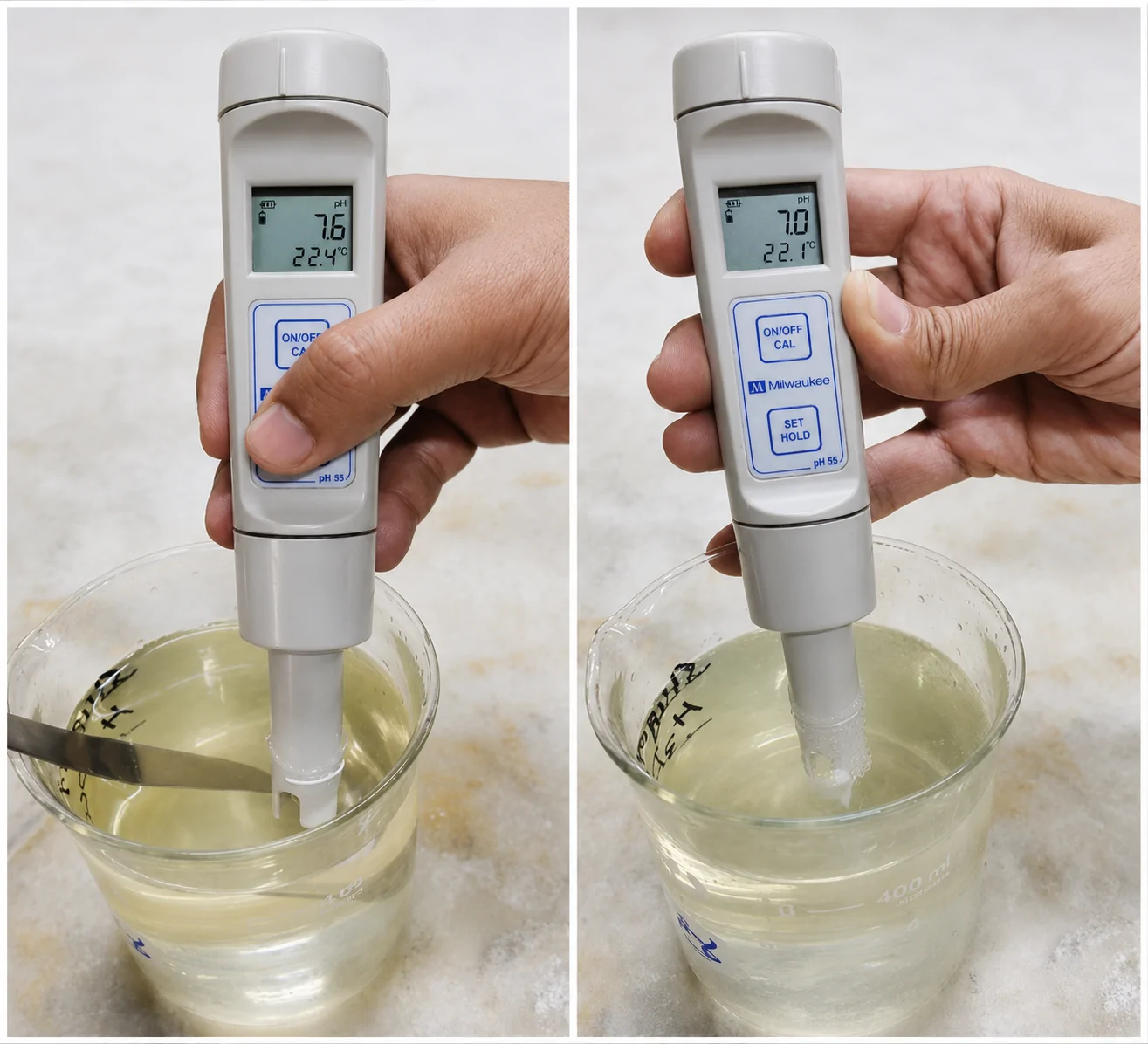
Measurement of pH value for extracts.

Each 10-gram soil sample was mixed with 20 grams of AB-DTPA solution with adjusted pH, and then the mixture was placed on a shaker and agitated for 20 minutes at a speed of 190 rpm to ensure optimal extraction of nutrients, as shown in Fig 4.

**Fig 4:**
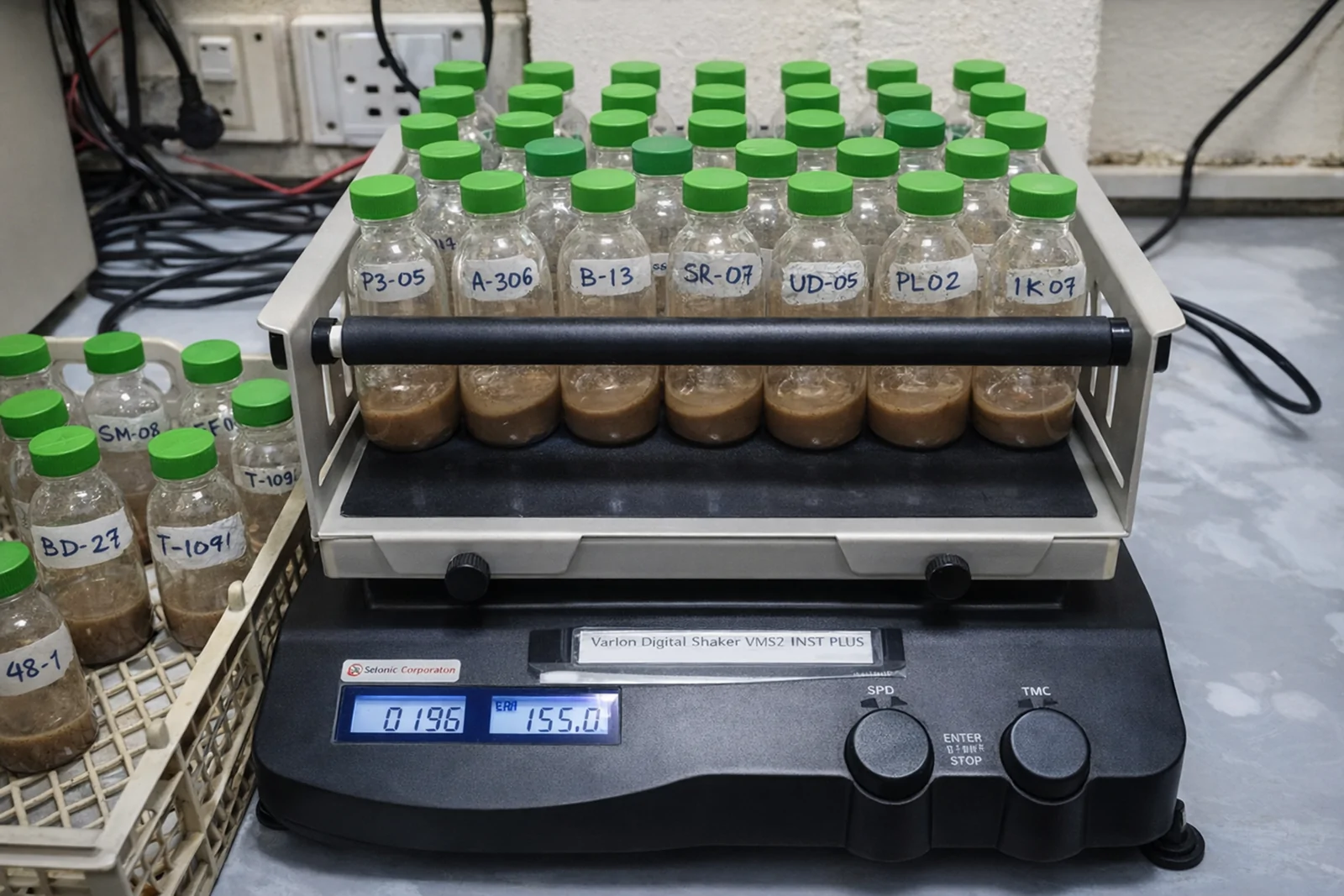
Digital Shaker for mixing soil samples.

The experiment was carried out for 20 minutes on a shaker. Then the extract was filtered through filter paper. This process was performed to remove solid particles, which yielded a clear solution suitable for nutrient analysis. The resulting filtrate was collected in a clean, transparent bottle. This step is very important as it ensured that the sample was properly collected and handled for accurate analysis. Any contamination or delay during the collection process can badly affect the quality and reliability of the results obtained from the subsequent analysis; this is shown in Fig 5.

**Fig 5:**
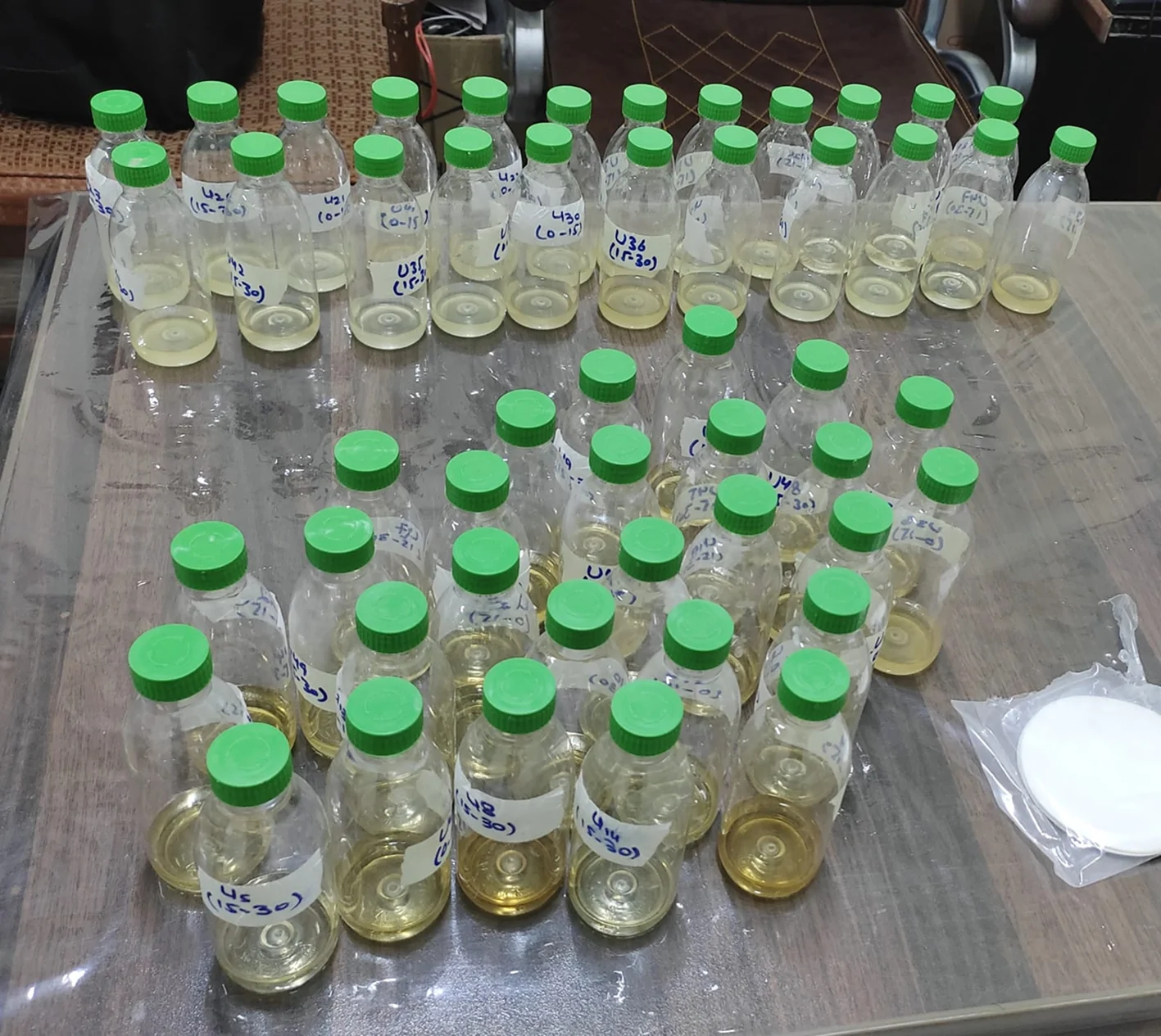
Final soil extract after processing.

### Preparation of stock solutions for nitrogen

To quantify nitrogen in soil extracts, four solutions were prepared:

- Copper Sulphate
- Sodium Hydroxide
- Hydrazine Sulphate
- Color Developer

### Nitrogen measurement procedure

- 1 mL of extract mixed with 3 mL CuSO_4_, 2 mL hydrazine sulfate, 3 mL NaOH.
- Heated at 38°C for 20 minutes, then 3 mL of color developer added.
- The solution turned pink.

The absorbance of the spectrophotometer was measured at 540  nm. A calibration curve was plotted to find NO_3_-N concentration.

### Phosphorus color reaction and measurement

- 0.739 g of L-ascorbic acid was incorporated into 140 mL of freshly prepared phosphorus reagent.
- Dilute 1  mL of soil extract with 10  mL of deionized (DI) water, then mix with 2.5  mL of the color reagent.
- Measure the absorbance at 880  nm after a reaction time of 30 minutes, as shown in Fig 6.

**Fig 6:**
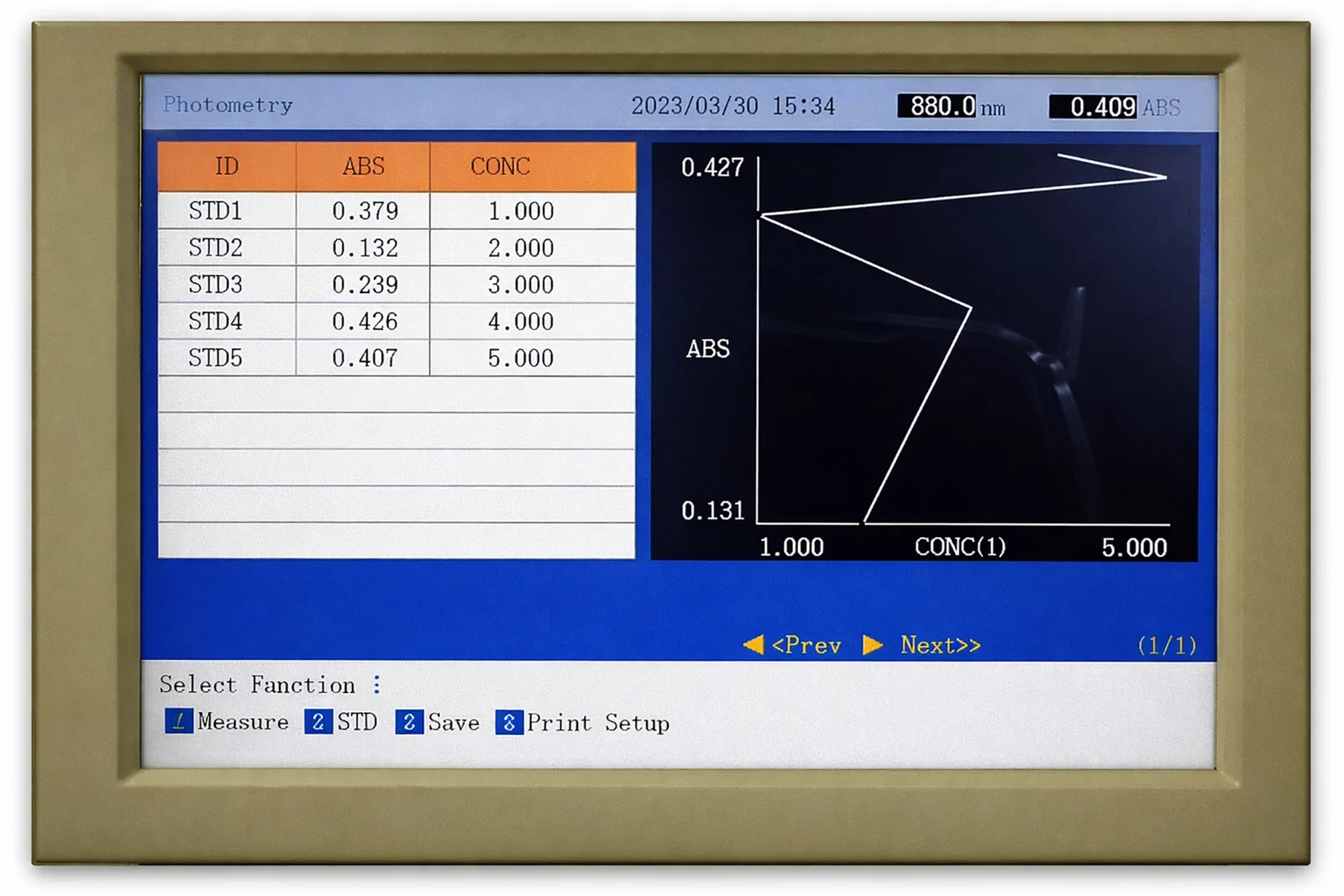
Spectrophotometer reading for phosphorus analysis.

### Potassium reagents and standards

The potassium measurement involved two key solutions:

1. **Ammonium Acetate:** Combine 57 mL of concentrated acetic acid with 68 mL of ammonium hydroxide in 800 mL of deionized (DI) water. Adjust the pH to 7.0, then make up the volume to 1  L.
2. **Potassium Chloride(KCl) Stock Solution:** 1.907 g of dried KCl, previously heated at 120 °C, was dissolved in DI water and diluted to 1 L, yielding a 1000 ppm stock solution.

To prepare standard solutions (40–200  ppm), varying volumes of the stock solution between 2 and 10  mL, with 2  mL increments, were diluted to 100  mL using 1  N ammonium acetate as shown in Fig 7.

**Fig 7:**
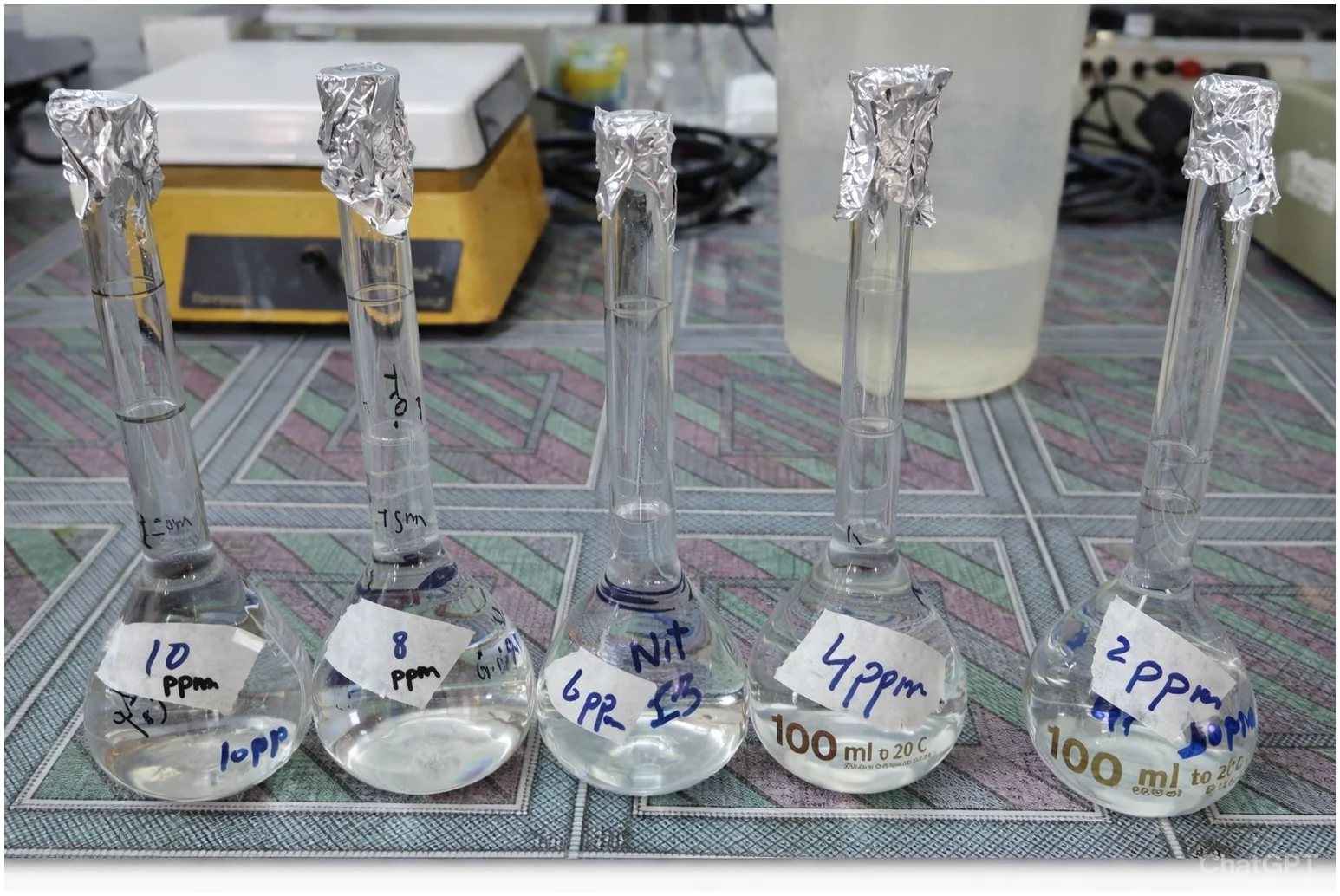
Preparation of potassium standard solutions.

### Potassium measurement procedure

A 1   mL portion of soil extract was mixed with 9   mL of DI water using a syringe and transferred to a clean container for analysis. The flame photometer was calibrated using the prepared potassium standards. The instrument’s intake tube was then immersed in the diluted soil extract. Flame intensity readings were recorded and compared with the calibration curve to determine potassium concentration as shown in Fig 8.

**Fig 8:**
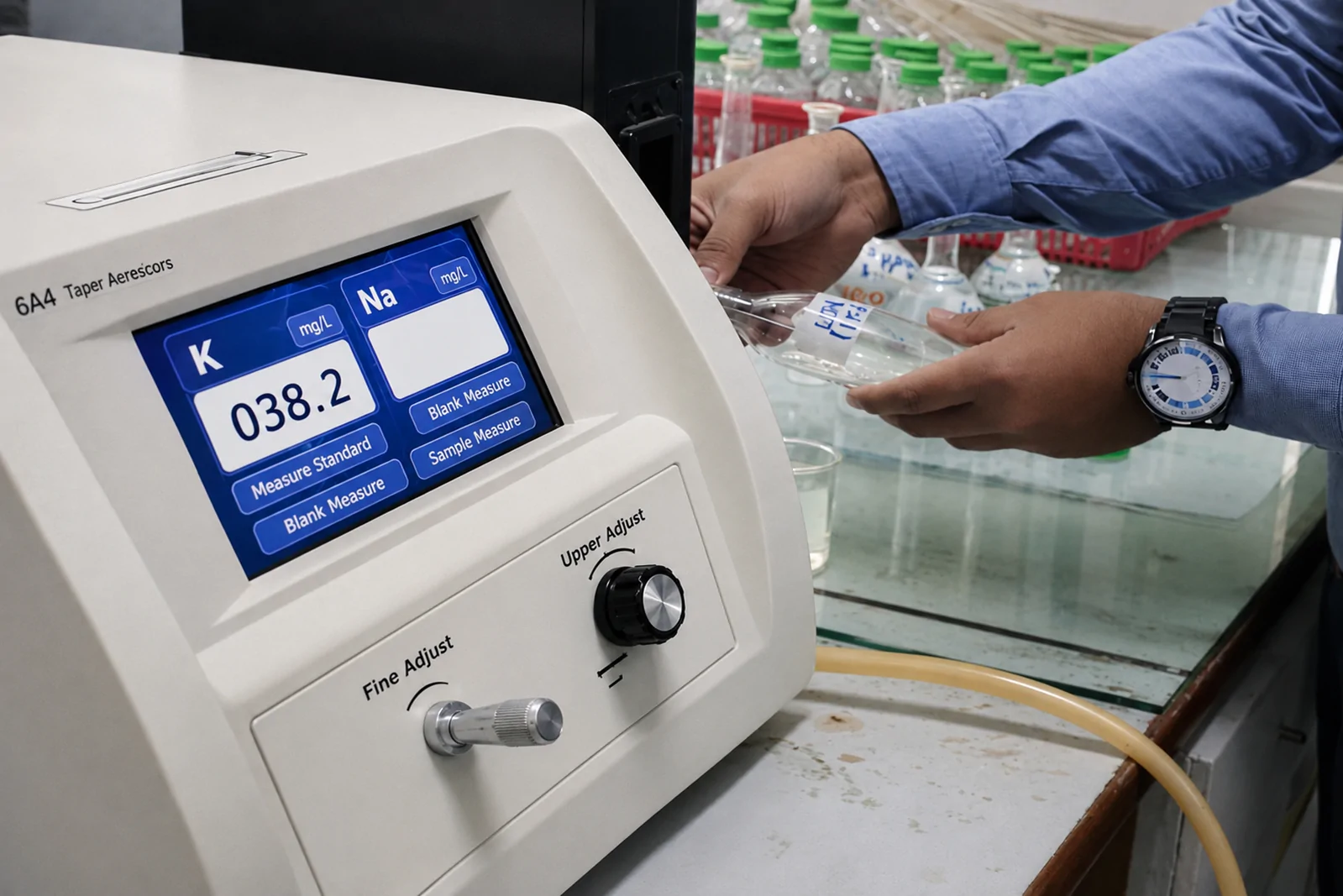
Measuring potassium using a flame photometer.

### Nutrient-class assignment

The laboratory-measured nutrient concentrations were converted into Low, Medium, and High classes using the operational thresholds applied in this study. The following Table 2 presents the threshold range and observed class distribution.

**Table 2:** Operational thresholds and observed class distribution for NPK status classification.

Nutrient	Low	Medium	High	Class counts (L/M/H)
Nitrogen	< 0.12	0.12– < 0.20	≥0.20	1/18/31
Phosphorus	< 0.50	0.50– < 2.50	≥2.50	41/6/3
Potassium	< 5.0	5.0– < 12.0	≥12.0	6/22/22

### Controlled image acquisition and processing

In order to reduce chemical dependency and develop a faster prediction mechanism, an image based prediction model for NPK classification was implemented.

#### Black box imaging setup.

To ensure consistent and interference-free imaging of soil extracts, a custom-built black-box imaging chamber was developed. 50 soil extract samples were placed in a custom-built black-box imaging chamber and photographed under the same lighting conditions. This controlled setup was designed to minimize the effect of environmental light fluctuations on image quality. The illumination level in lux was monitored during image acquisition with a Mini Light Meter UNI-T UT383. The captured pictures were saved in a series of images from U1.jpg to U50.jpg.

#### Black-box design for image processing.

Natural or artificial lighting changes may have a great impact on the reliability of image-based analysis. So, a controlled imaging enclosure (referred to as a black box) was constructed using 3 mm-thick plastic glass. The standardized size of the box was 1.5 feet per side. It has a removable lid made from the same material to ensure a tight fit over the enclosure. The edges of the lid were bent inward to prevent external light from entering the chamber, as illustrated in Fig 9.

**Fig 9:**
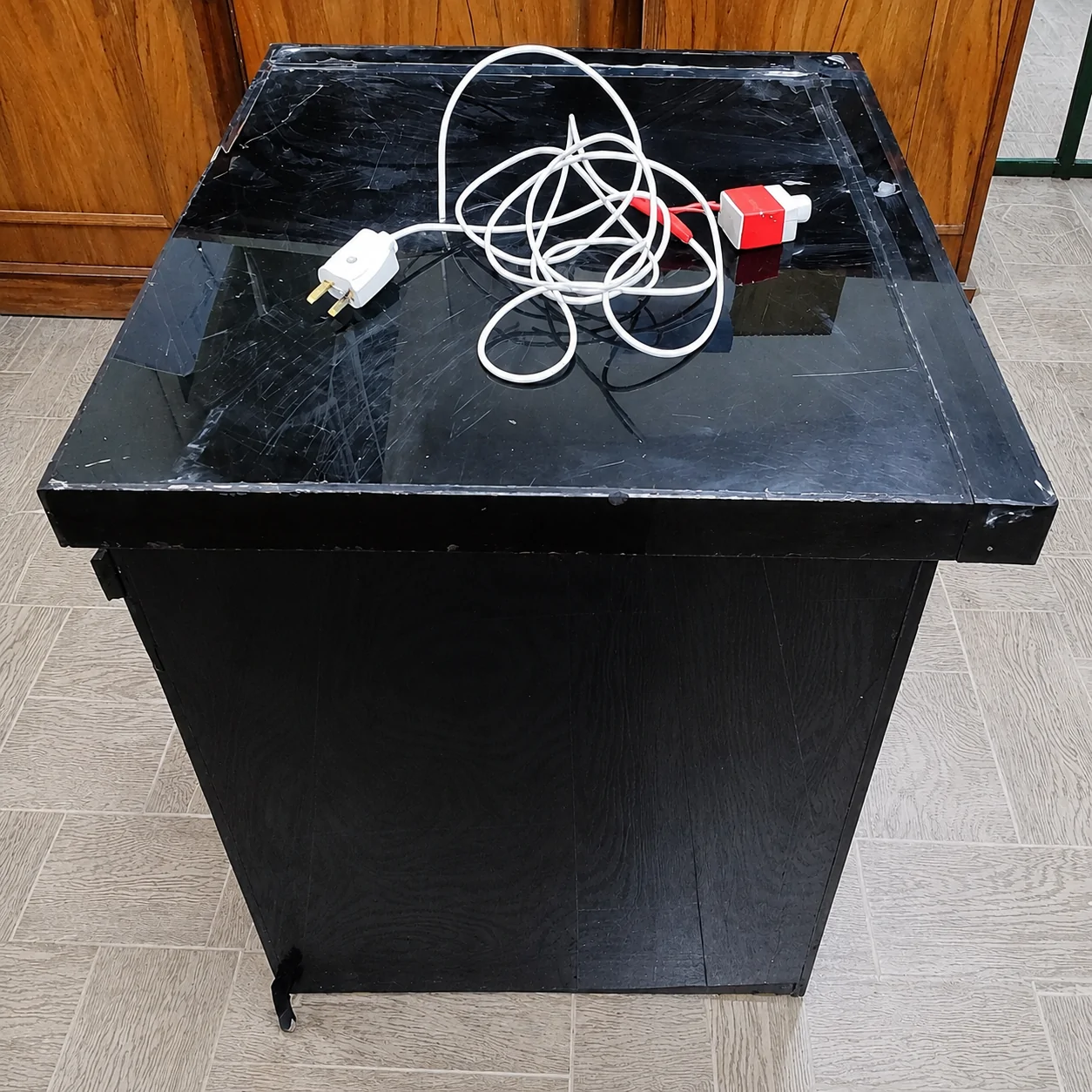
Black box prototype.

#### Hardware components of the black box.

The setup consisted of many key components such as the base platform, sample holder, illumination system, light monitoring, and camera placement. Each of these was designed to ensure efficient and reliable image acquisition, with full details provided in Table 3.

**Table 3:** Setup of black box for image acquisition.

Component	Description
Base Platform	A plain white sheet was used to minimize glare and provide a neutral background for consistent RGB extraction.
Sample Holder	Soil extracts were placed in white ceramic dishes to enhance contrast and improve image clarity.
Illumination System	A 12 W white LED bulb was installed inside the lid to provide uniform lighting. Brightness was controlled using a fan regulator.
Light Monitoring	A lux meter was used to ensure consistent light intensity during image capture.
Camera Placement	A fixed mobile holder ensured stable positioning. A Xiaomi Redmi Note 10 (48 MP, f/1.8 wide-angle lens) was used for capturing images.

By maintaining fixed lighting conditions, this configuration standardized image acquisition and reduced variability in the extracted features. The imaging setup of the black box is shown in Fig 10.

**Fig 10:**
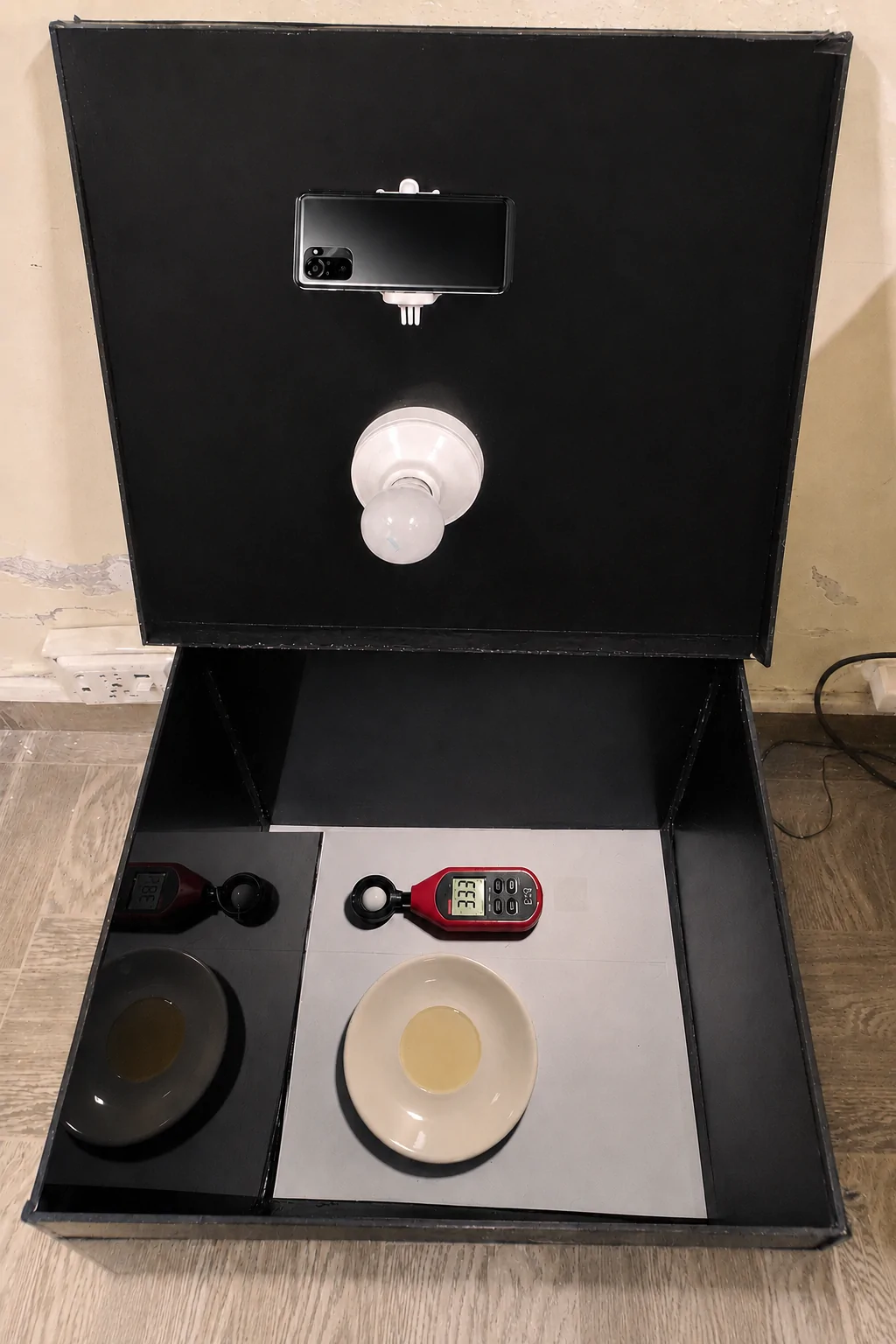
Setup of black box for image acquisition.

### Data splitting, image preprocessing, feature extraction, and augmentation

The dataset consisted of 50 independent physical soil samples. Using those samples, images of their extracts were taken under the same lighting conditions in the black-box imaging box. In the final classical machine-learning analysis, for N, P, and K, five outer sample-level folds were used, while for hyperparameter selection, three-fold stratified cross-validation was performed in each outer training fold. The partitioning of the dataset was performed at the physical-sample level before feature extraction, augmentation, and model training so that each held-out physical sample was independent of the training data.

#### Image preprocessing.

In each soil extract image, the region containing the liquid soil extract was cropped while excluding the ceramic dish rim and surrounding background. Before feature extraction and CNN training, the selected region was resized uniformly; the images were kept in RGB format and later converted to HSV for color feature extraction. In CNN training, pixel values were normalized before being provided to the network. The images were taken with controlled and consistent lighting inside the black-box chamber, so no additional denoising filter was applied.

#### Feature extraction for machine learning.

In the classical machine-learning analysis, the central 50% region of each soil-extract image was cropped and resized to 224 × 224 pixels before feature extraction, while for each image, a total of 185 color and texture descriptors were extracted. Color information consisted of statistical descriptors derived from RGB, HSV, CIELAB, and YCbCr color spaces together with grayscale statistics, normalized RGB chromaticity components, and inter-channel color ratios. Various statistics, such as minimum, maximum, median, standard deviation, and selected percentiles, were calculated for the applicable image channels. To extract texture and structural information, the grayscale entropy, Sobel edge statistics, Local Binary Pattern (LBP) features, and Gray-Level Co-occurrence Matrix (GLCM) properties such as contrast, dissimilarity, homogeneity, energy, correlation, and angular second moment were used. All non-finite numerical values generated during feature computation were replaced with finite values before model fitting.

#### Image augmentation for CNN training.

The CNN training images were only augmented after the outer-fold split on physical samples, and augmentation was only applied to the training data. The horizontal and vertical flips, random rotations up to ±7°, and the zoom transformations were applied dynamically during model training. These augmented images were treated only as transformed training variants and were not considered additional independent observations. No augmentation was applied to the held-out outer-fold test images. Brightness and color transformations were not used, as visible color was considered an important analytical signal for nutrient-status classification. The CNN evaluation was performed without any test-time augmentation.

#### Leakage prevention.

Augmented versions of validation or test samples were never included in the training data. Brightness and color transformations were not included since visible color was the main analytical signal, and on test images no augmentation was applied.

#### CNN feature learning.

The CNN automatically learned image features directly from cropped and resized RGB images received by the CNN. Therefore, the classical ML models were only used with manually calculated RGB, HSV, and texture descriptors, which were not provided as CNN inputs. The summary of the model evaluation pipeline is described in Table 4.

**Table 4:** Summary of the leakage-controlled image-processing and model-evaluation pipeline.

Step	Description
Image Capture	Fifty independent soil-extract samples were imaged under controlled lighting in a black-box environment.
Dataset Splitting	Physical samples were partitioned into five leakage-controlled outer folds that were used consistently for nitrogen, phosphorus, and potassium evaluation.
Region of Interest (ROI) Cropping and Preprocessing	The liquid-extract region was cropped, the dish rim and surrounding background were excluded, and the images were resized uniformly.
Feature Extraction	RGB, HSV, and texture descriptors were extracted for the classical machine-learning models.
Data Augmentation	Training-only geometric augmentation was applied to the CNN images; the number of independent physical samples remained 50.
Label Assignment	Samples were assigned Low, Medium, or High classes according to the operational thresholds reported in the nutrient-classification table.
Model Training and Evaluation	Classical machine-learning models and a CNN were evaluated using out-of-fold predictions, accuracy, balanced accuracy, macro-F1 score, Cohen’s kappa, MCC, confidence intervals, majority-class baselines, and confusion matrices.

### ML and DL model development

After image preprocessing and data preparation, separate models were developed for the classification of nitrogen status, phosphorus status, and potassium status. The machine learning algorithms used for supervised learning that were evaluated were: Random Forest, Support Vector Machine, Gaussian Naive Bayes, Gradient Boosting, K-Nearest Neighbours, and Logistic Regression. The extracted RGB, HSV, and texture features were used as inputs to these models. Furthermore, a CNN was trained directly from cropped and resized RGB images to perform automatic feature learning and classification. Seven models were evaluated for the classification of each nutrient into Low, Medium, or High. The classical machine-learning and CNN models were implemented in Python. The analysis notebooks were verified using Python 3.13.15, NumPy 2.1.3, pandas 2.2.3, scikit-learn 1.6.1, scikit-image 0.25.2, and Pillow 11.3.0.

### Classical ML models

The classical ML models were tested by the leakage-controlled nested cross-validation approach at the physical-sample level. The five outer folds were used for held-out sample-level predictions, and the three-fold stratified cross-validation was used in each outer training fold for hyperparameter selection. Only samples in the same training fold were used for training data augmentation. The original images and mild transformed images were used for feature extraction and model fitting, with a total of 10 image representations per physical training sample. The hyperparameter settings were prioritized mainly by the inner-fold macro-F1 score, followed by balanced accuracy, Cohen’s kappa, and accuracy. Feature preprocessing, feature selection, scaling, and model fitting were performed only using the corresponding training data.

Training-data augmentation involved horizontal mirroring, rotations within ±7°, zooming from 1.00 to 1.08, and mild brightness and contrast changes within 0.96 and 1.04. These transformed images were generated using only training-fold samples and were not treated as independent physical observations.

Additionally, in the outer-fold evaluation, the test-time augmentation (TTA) was used to ensure the stability of the prediction of each held-out physical sample. Five representations were tested for each test sample, including the original image and four mild image variants. They were aggregated to create a single sample-level prediction, and their transformed variables were not treated as additional test observations.

A fixed random seed of 42 was used to ensure reproducibility of the fold assignments and model training. Stratified evaluation with multiple random seeds was not applied as the number of samples per class was very small, especially for the minority Low-nitrogen sample and three High-phosphorus samples, which prevented reliable class representation across repeated stratified folds; therefore, uncertainty was reported using sample-level out-of-fold predictions and 95% confidence intervals.

To increase the reproducibility of the classical machine-learning pipelines, median imputation, removal of zero-variance features, and univariate feature selection based on the ANOVA F-statistic were performed. Only the inner-fold cross-validations were used for hyperparameter selection, while KNN, Logistic Regression, and Gaussian Naive Bayes were applied using standard scaling, and robust scaling was used for the RBF-SVM. The set of candidate hyperparameter settings evaluated in the inner-fold model selection is summarized in Table 5.

**Table 5:** Hyperparameter search space used for the classical machine-learning models.

Model	Hyperparameters evaluated / fixed settings
KNN	Selected feature percentile: 15, 30, 50%; neighbors: 3, 5, 7, 9; distance weighting; Minkowski distance with *p* = 2
SVM-RBF	Selected feature percentile: 15, 30, 50%; *C*: 0.5, 2.0, 8.0; RBF kernel; γ=scale; class weight = balanced
Logistic Regression	Selected feature percentile: 15, 30, 50%; *C*: 0.1, 1.0, 10.0; class weight = balanced; maximum iterations = 5000
Random Forest	Selected feature percentile: 30, 50, 100%; 300 trees; max features = square root; minimum samples per leaf: 1 or 2; class weight = balanced subsample
Extra Trees	Selected feature percentile: 30, 50, 100%; 300 trees; max features = square root; minimum samples per leaf: 1 or 2; class weight = balanced
Gradient Boosting	Selected feature percentile: 30 or 50%; 150 estimators; learning rate: 0.03 or 0.07; maximum depth: 1 or 2
Gaussian Naive Bayes	Selected feature percentile: 15, 30, 50%; variance smoothing: 10^–9^, 10^–8^, 10^–7^

### CNN architecture and training

A compact CNN network was trained directly on cropped RGB images, while the central 50% region of each image was extracted and cropped to 224 × 224. The network was composed of three convolutional layers: 12, 24, and 48 filters, ReLU activation functions, and a kernel size of 3x3. Max-pooling, global average pooling, a dropout probability of 0.25, and a softmax output layer with three classes were used. The network was trained with an Adam optimizer with a learning rate of 0.002, sparse categorical cross-entropy loss, a batch size of 8, a maximum of 25 epochs, and early stopping with a patience of 5 epochs. A fixed random seed of 42 was also used for reproducibility.

The performance of the CNN was evaluated with the same five physical sample folds used for the classical machine-learning analysis. Within each outer training fold, approximately 20% of the training samples were separated for early stopping, while the outer held-out samples remained completely independent. The augmentation of the images was performed only during the training of the CNN and included horizontal and vertical flipping, rotation up to about ±7°, and zoom up to 8%. No brightness or color changes were made. No augmentation or test-time augmentation was applied to the held-out outer-fold images. Final CNN performance was calculated from the pooled out-of-fold predictions of all 50 independent physical samples.

### Performance evaluation metrics

Standard classification metrics were calculated to evaluate model performance in more detail: Precision, Recall, F1-Score, and Accuracy for each class (Low, Medium, High) of NPK. These metrics are especially useful when there is class imbalance and can provide a detailed explanation of the prediction results. Precision is the ratio of $T_{p}$ cases to the number of cases predicted as positive and may be represented by:


$$\begin{array}{c} \text{Precision}=\frac{T_{p}}{T_{p}+F_{p}} \end{array}$$ (1)

here, $T_{P}$ denotes all true positives values and *FP* all false positives values [31]. Recall is a measure of how many of the actual positives the model correctly identifies and is represented as:


$$\begin{array}{c} \text{Recall}=\frac{T_{p}}{T_{p}+F_{n}} \end{array}$$ (2)

where $F_{n}$ represents the number of false negative tests [32]. The balance measure for model performance is provided by using the F1 Score, which is derived by taking the harmonic mean of precision and recall, as given in equation (3)


$$\begin{array}{c} \text{F1\{Score}\}=\frac{2\times (\text{Precision}\times \text{Recall})}{\text{(}Precision+\text{Recall})} \end{array}$$ (3)

While handling an imbalanced dataset, F1-Score plays a significant role in testing the performance of models[33].. Another popular evaluation measure is Accuracy, which is the percentage of the samples for which the prediction was correct out of all predictions [34].


$$\begin{array}{c} \text{Accuracy}=\frac{T_{p}+T_{n}}{T_{p}+T_{n}+F_{p}+F_{n}} \end{array}$$ (4)

Along with the accuracy, precision, recall, and F1 score, the final sample-level evaluation consists of balanced accuracy, macro-F1, weighted-F1, Cohen’s kappa, and MCC. MCC was added as another imbalance-aware measure because it considers the overall relationship between the observed and predicted classes. Accuracy uncertainty was summarized using 95% Wilson confidence intervals based on the pooled out-of-fold predictions.

The out-of-fold correctness outcomes were compared pairwise statistically with the exact McNemar test on the sample level between the selected classical model and the alternative classical models. Because multiple model comparisons were conducted within each nutrient target, p-values were adjusted using the Holm procedure, with an adjusted *p* < 0.05 considered statistically significant.

## Results

The final evaluation was performed on 50 independent physical soil samples. Derived image variants were included only in the training data and were not counted as additional independent observations, and no single 80:20 train-test split was used in the final evaluation for the classical machine-learning analysis. Rather, a leakage-controlled nested sample-level cross-validation, with five outer folds and three inner folds, was used, with a fixed random seed of 42. The inner folds were used to apply hyperparameter tuning, while the outer folds were used to produce out-of-fold predictions for the independent physical samples. The CNN was validated separately by leakage-controlled out-of-fold validation with augmentation restricted to the corresponding training data

### Descriptive statistics and nutrient variability

The descriptive statistics of the laboratory-measured nutrient concentrations used as reference observations for the classification analysis are shown in Table 6. Nitrogen ranged from 0.119 to 1.694, with a mean of 0.386 and a standard deviation (SD) of 0.341. Phosphorus ranged from 0.207 to 2.836, with a mean of 0.495 and an SD of 0.596, while potassium ranged from 2.900 to 28.500, with a mean of 11.862 and an SD of 5.553.

**Table 6:** Descriptive statistics of the laboratory-measured nutrient concentrations for the 50 independent soil samples.

Nutrient	n	Minimum	Maximum	Mean	SD	Median	CV (%)
Nitrogen	50	0.119	1.694	0.386	0.341	0.234	88.22
Phosphorus	50	0.207	2.836	0.495	0.596	0.297	120.36
Potassium	50	2.900	28.500	11.862	5.553	11.600	46.81

The relative variability was highest for phosphorus with a coefficient of variation of 120.36%, followed by nitrogen with a CV of 88.22% and potassium with a CV of 46.81%, as shown in Fig 11.

**Fig 11:**
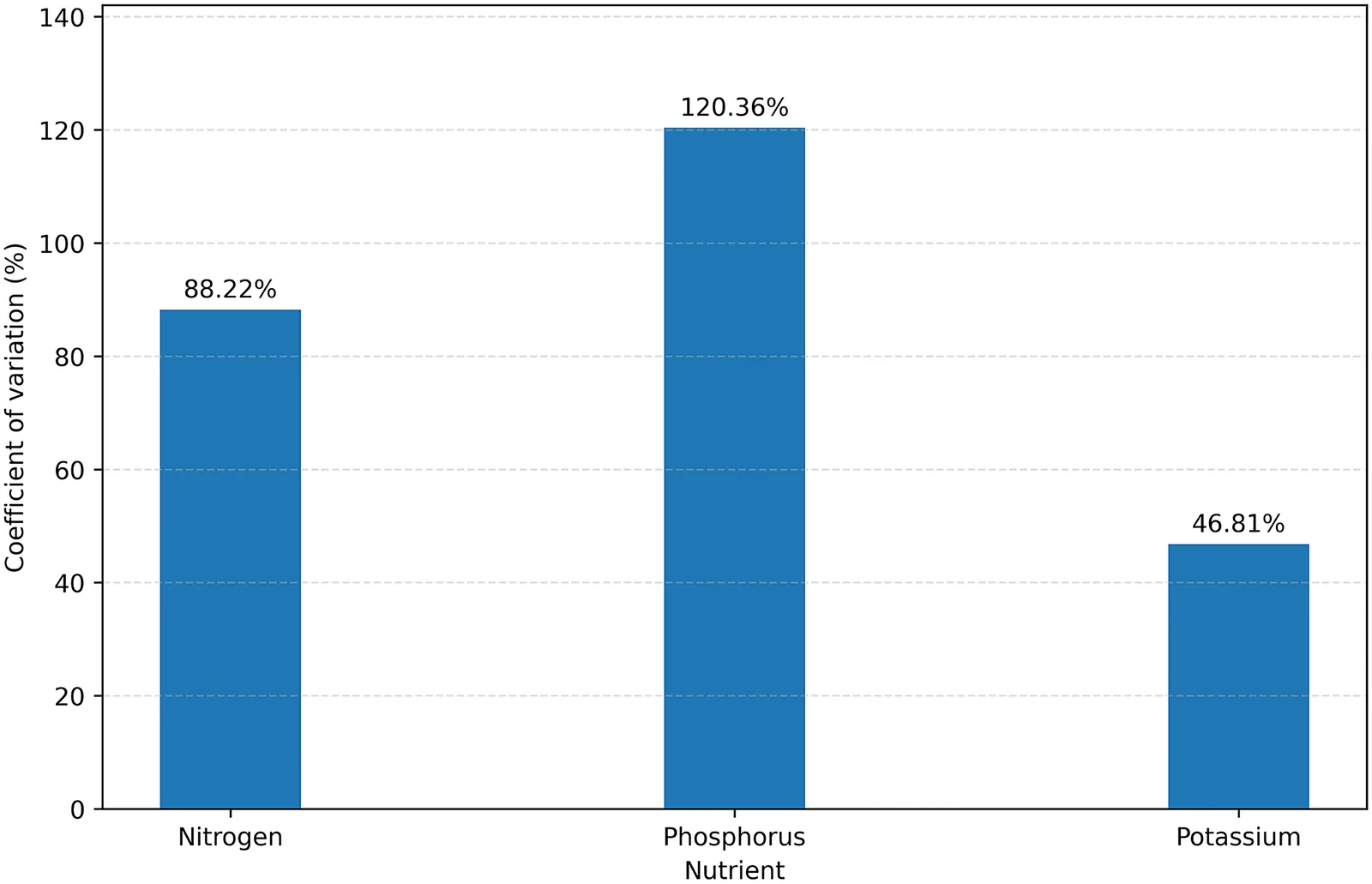
Relative variability of laboratory-measured NPK concentrations in the 50 independent soil samples.

### Nutrient class distribution

The class distributions were very imbalanced for both nitrogen and phosphorus, as shown in Fig 12 and summarized in Table 7. One sample was labelled as Low, 18 as Medium, and 31 as High for nitrogen. The distribution of phosphorus samples was: Low = 41, Medium = 6, and High = 3. Potassium was relatively balanced, with six Low, 22 Medium, and 22 High samples. The majority of class baselines were 62%, 82%, and 44% for N, P, and K, respectively.

**Fig 12:**
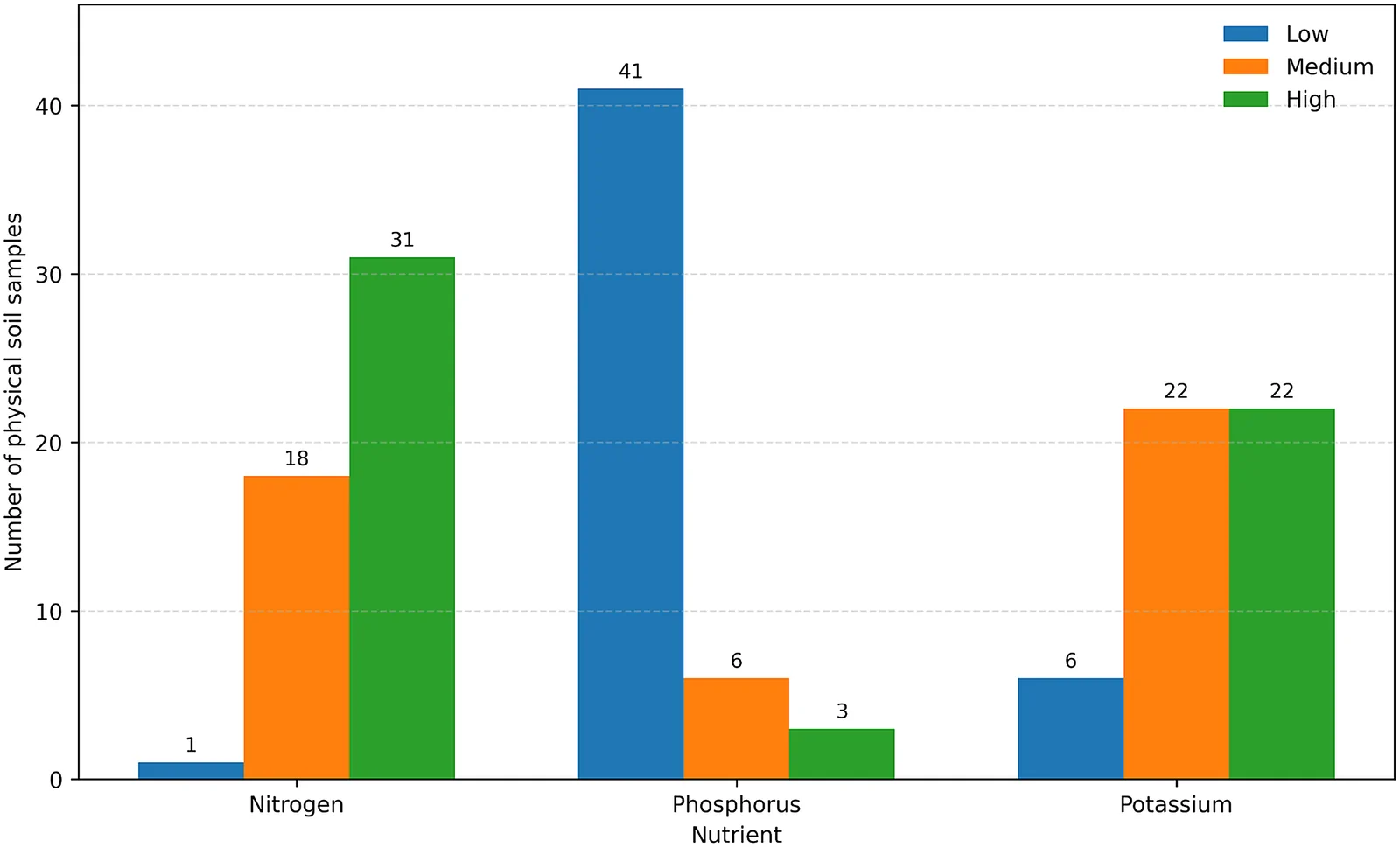
Distribution of low, medium, and high nutrient classes among the 50 independent physical soil samples.

**Table 7:** Distribution of low, medium, and high nutrient classes.

Nutrient	Low	Medium	High	Majority baseline
Nitrogen	1 (2%)	18 (36%)	31 (62%)	62%
Phosphorus	41 (82%)	6 (12%)	3 (6%)	82%
Potassium	6 (12%)	22 (44%)	22 (44%)	44%

### Observed concentration ranges within the assigned classes

The observed ranges for the concentrations in the labels contained in the data set are summarized in Table 8. These values represent the values of the current sample set.

**Table 8:** Observed concentration ranges within the assigned nutrient classes.

Nutrient	Class	Samples	Observed minimum	Observed maximum
Nitrogen	Low	1	0.119	0.119
	Medium	18	0.122	0.199
	High	31	0.220	1.694
Phosphorus	Low	41	0.207	0.484
	Medium	6	0.548	1.042
	High	3	2.661	2.836
Potassium	Low	6	2.900	4.800
	Medium	22	5.800	11.900
	High	22	12.200	28.500

### Nested cross-validation results of the classical ML models

The final classical ML results were obtained from the inner-fold hyperparameter search and selected by pooled outer-fold macro-F1 score, as shown in Fig 13.

**Fig 13:**
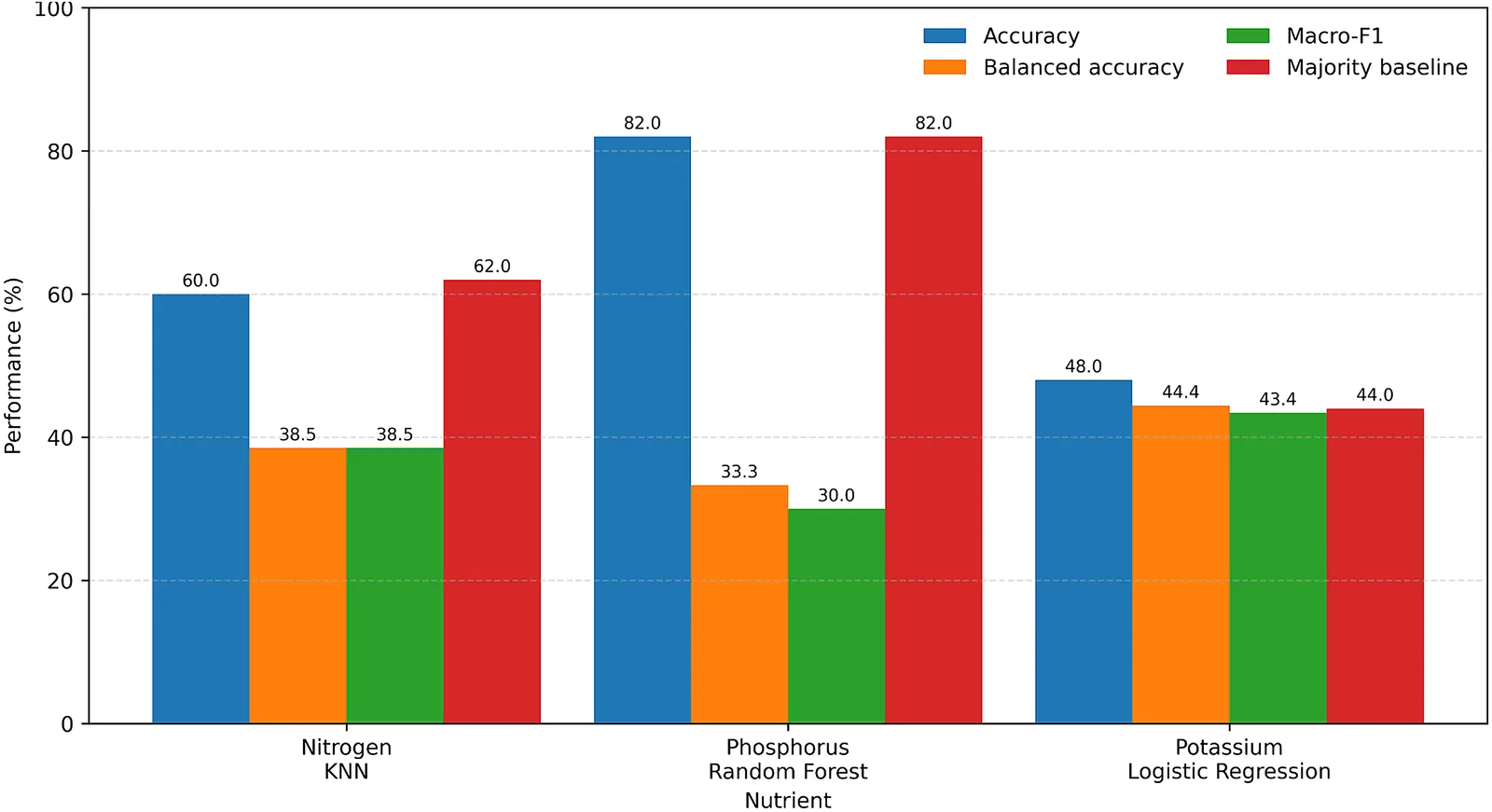
Performance of selected classical ML models and the majority-class baseline under nested sample-level cross-validation.

By comparing the results of these models with each other, as presented in Table 9, KNN achieved an accuracy of 60% for nitrogen, Random Forest achieved 82% for phosphorus, and Logistic Regression achieved 48% for potassium. The KNN accuracy for nitrogen was slightly lower than the majority baseline of 62%. The macro-F1 score is 0.385, and its balanced accuracy is 38.5%, indicating that classification performance differed across classes. The model mainly classified the Medium and High classes; the single Low nitrogen sample was not classified correctly.

**Table 9:** Final nested cross-validation results of the selected classical machine-learning models.

Nutrient	Selected model	Accuracy	95% CI	Balanced acc.	Macro-F1	Weighted-F1	MCC	Kappa	Baseline
Nitrogen	KNN	0.600	0.462–0.724	0.385	0.385	0.598	0.168	0.167	0.620
Phosphorus	Random Forest	0.820	0.692–0.902	0.333	0.300	0.739	0.000	0.000	0.820
Potassium	Logistic Regression	0.480	0.348–0.615	0.444	0.434	0.487	0.159	0.158	0.440

In the case of phosphorus, Random Forest reached 82% accuracy, which was the same as the 82% majority-class baseline. A confusion matrix revealed that all samples were predicted as Low, resulting in correct classification only for the samples whose actual class was Low. As a result, the model achieved zero recall for both the Medium and High classes and a balanced accuracy of 33.3% and a Cohen’s kappa of 0.000.

For potassium, Logistic Regression achieved 48% accuracy, four percentage points higher than the 44% majority baseline. Its balanced accuracy was 44.4%, macro-F1 was 0.434, and Cohen’s kappa was 0.158. This is the most balanced of the selected ML results, but the performance remained modest.

After Holm correction, no statistically significant differences were found between the selected model and the alternative classical models for nitrogen or potassium using pairwise exact McNemar tests. Random Forest also achieved a significantly higher paired accuracy than Gaussian Naive Bayes (Holm-adjusted p = 0.0312), Gradient Boosting (p = 0.0195), and Logistic Regression (p = 0.0117), but no significant difference was observed from Extra Trees, KNN, and RBF-SVM. These results should be interpreted along with the imbalance-aware measurements, as the overall accuracy of the phosphorus model was 82%, but its balanced accuracy was 0.333, and MCC was 0.000.

### Ablation analysis

A controlled ablation analysis was performed to investigate the contribution of various handcrafted feature groups and augmentation strategies, while keeping the same physical-sample outer folds and selected model families from the primary analysis. The feature ablations were compared among RGB-only, HSV-only, combined RGB–HSV, and the full representation of 185 features, as summarized in Table 10.

**Table 10:** Feature ablation results for the selected classical machine-learning models.

Nutrient	Model	Feature condition	Accuracy	Balanced accuracy	Macro-F1	MCC
Nitrogen	KNN	RGB only	0.52	0.295	0.272	−0.141
		HSV only	0.50	0.300	0.292	−0.107
		RGB + HSV	0.56	0.324	0.306	−0.035
		Full feature set	**0.60**	**0.385**	**0.385**	**0.168**
Phosphorus	Random Forest	RGB only	0.82	0.333	0.300	0.000
		HSV only	0.82	0.333	0.300	0.000
		RGB + HSV	0.82	0.333	0.300	0.000
		Full feature set	0.82	0.333	0.300	0.000
Potassium	Logistic Regression	RGB only	**0.52**	**0.475**	**0.468**	**0.290**
		HSV only	0.26	0.237	0.234	−0.123
		RGB + HSV	0.32	0.283	0.283	−0.048
		Full feature set	0.48	0.444	0.434	0.159

The three conditions were considered for the augmentation ablation: no augmentation, training-only augmentation, and training augmentation with test-time augmentation (TTA), which are summarized in Table 11. The same modeling configurations as the main analysis were used for each fold to assess the effect of the ablated component under otherwise identical modeling conditions.

**Table 11:** Augmentation ablation results using the full handcrafted feature set.

Nutrient	Model	Augmentation condition	Accuracy	Balanced accuracy	Macro-F1	MCC
Nitrogen	KNN	No augmentation	0.56	0.332	0.321	−0.007
		Training augmentation only	**0.64**	**0.414**	**0.415**	**0.251**
		Training augmentation + TTA	0.60	0.385	0.385	0.168
Phosphorus	Random Forest	No augmentation	0.82	0.333	0.300	0.000
		Training augmentation only	0.82	0.333	0.300	0.000
		Training augmentation + TTA	0.82	0.333	0.300	0.000
Potassium	Logistic Regression	No augmentation	0.36	0.354	0.343	−0.047
		Training augmentation only	0.46	0.429	0.414	0.127
		Training augmentation + TTA	**0.48**	**0.444**	**0.434**	**0.159**

The individual contributions of feature groups and augmentation strategies across nutrients are shown in Tables 10 and 11. The findings from the ablation indicated that the individual components differed across nutrients. In the case of nitrogen, the feature-based approach was the best with full handcrafted feature representation, while the training-only augmentation was better than no augmentation and adding TTA. All ablation conditions yielded the same accuracy and imbalance-aware metrics for phosphorus, suggesting that the apparent 82% accuracy was due mainly to the large class size, rather than improved discrimination among nutrient classes. Training augmentation was effective, and even a little more effective when combined with TTA. These results suggest that both additional man-made structures and TTA were not uniformly beneficial to all nutrient targets.

### Machine-learning sensitivity analysis

Additionally, a sensitivity experiment was performed with the chosen machine-learning models using training-only augmentation while the held-out test samples did not change. Table 12 shows the performance of the ML models for nitrogen, phosphorus, and potassium.

**Table 12:** Performance of the selected ML Models in the training-only augmentation sensitivity experiment.

Nutrient	ML model	Accuracy	Balanced accuracy	Macro-F1	Baseline
Nitrogen	KNN	66%	40.9%	0.405	62%
Phosphorus	Logistic Regression	64%	30.8%	0.310	82%
Potassium	Random Forest	52%	43.4%	0.437	44%

For the three nutrients, namely N, P, and K, the KNN ML model gave the best accuracy of 66% for nitrogen, Logistic Regression 64%, and Random Forest 52%, respectively. The nitrogen and potassium models outperformed their majority class baselines, suggesting that the ML models had identified valuable information about the nutrients in soil images. The results of the nested cross-validation were kept as the main results, as they would give a better estimate of model generalization.

### Leakage-controlled compact CNN results

The compact CNN was tested using a pool of out-of-fold predictions from 50 independent physical samples. The accuracy of the compact CNN for nitrogen, phosphorus, and potassium was 58%, 82%, and 44%, respectively, as shown in Fig 14. Nitrogen was still four percentage points lower than the 62% majority-class baseline, while phosphorus and potassium were the same as their majority-class baselines of 82% and 44%, respectively.

**Fig 14:**
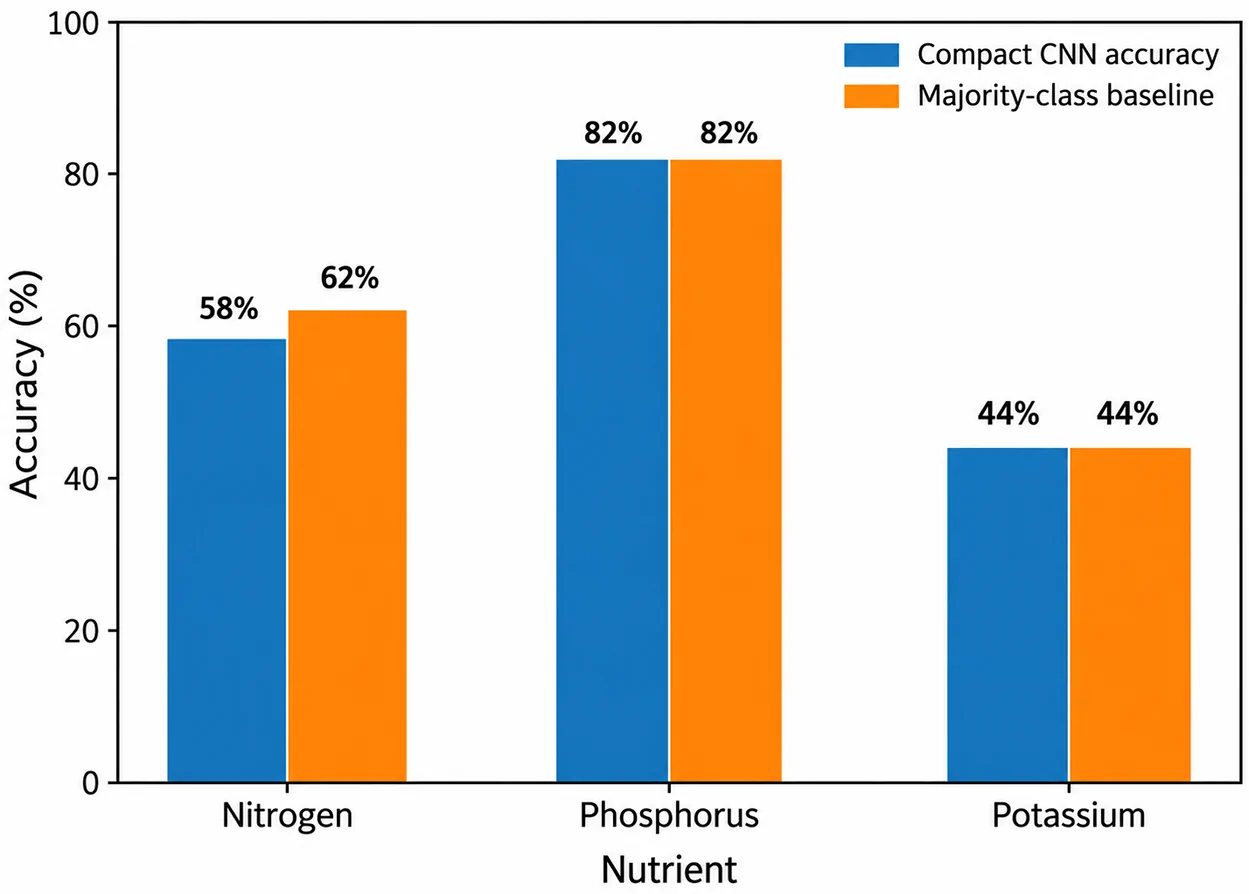
Compact CNN accuracy compared with majority-class baselines.

The Nitrogen CNN showed limited discrimination, with the balanced accuracy being 0.343 and MCC of 0.030. For phosphorus, the 82% accuracy matched the majority-class baseline, while balanced accuracy and MCC were 0.333 and 0.000, respectively, showing that the model was mainly representing the dominant low class. Similarly, the potassium CNN achieved balanced accuracy of 0.333 and MCC of 0.000 with the 44% majority class baseline, as summarized in Table 13. The results show that the compact CNN didn’t give a consistent predictive advantage under the present small and imbalanced dataset.

**Table 13:** Overall classification performance for nitrogen, phosphorus, and potassium.

Nutrient	Accuracy	95% CI	Balanced accuracy	Macro-F1	Weighted-F1	MCC	Cohen’s kappa	Majority baseline
Nitrogen	0.580	0.442–0.706	0.343	0.330	0.539	0.030	0.028	0.620
Phosphorus	0.820	0.692–0.902	0.333	0.300	0.739	0.000	0.000	0.820
Potassium	0.440	0.312–0.577	0.333	0.276	0.365	0.000	0.000	0.440

### Comparison of classical ML and deep-learning models

The proposed classical ML models and compact CNN were evaluated against nutrient-specific majority-class baselines as illustrated in Fig 15. The accuracy of KNN and CNN was 60% and 58%, respectively, for nitrogen, and both were lower than the 62% majority-class baseline value. For phosphorus, both Random Forest and the CNN achieved 82% accuracy, which was identical to the 82% majority-class baseline, while for potassium, Logistic Regression obtained an accuracy of 48%, which is slightly above the baseline of 44%, while the CNN matched the baseline at 44%. These results show that the compact CNN did not outperform the chosen classical models under the present small and imbalanced dataset.

**Fig 15:**
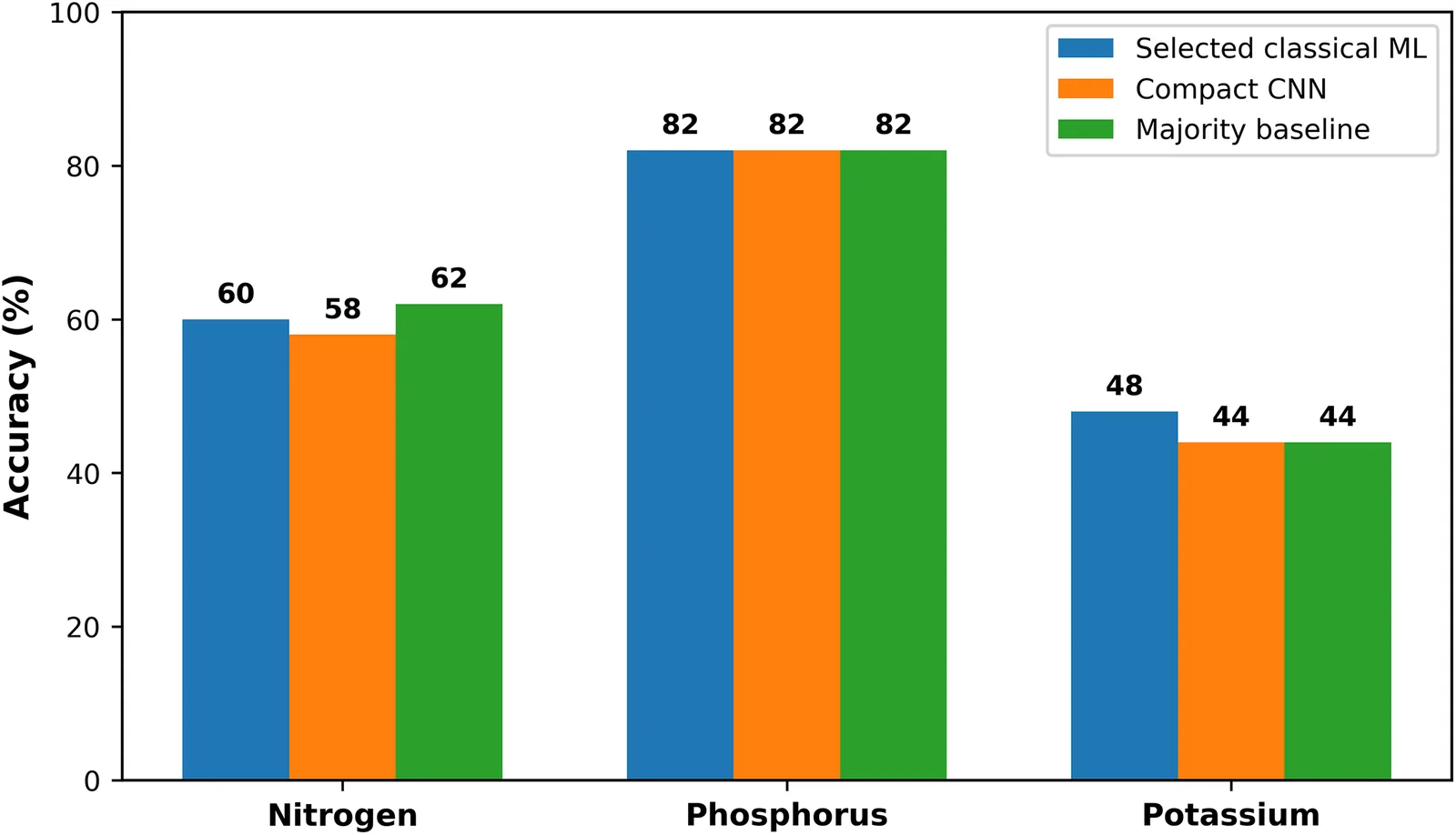
Accuracy comparison of the selected classical ML Models, compact CNN, and the nutrient-specific majority-class baselines.

### Confusion-matrix analysis

Model performances were evaluated using confusion matrices, with diagonal elements showing the number of correct predictions and off-diagonal elements showing the number of misclassifications for each class. The three ML confusion matrices for nitrogen, phosphorus, and potassium are plotted horizontally together in Fig 16, and the CNN confusion matrices for nitrogen, phosphorus, and potassium are plotted in Fig 17.

**Fig 16:**
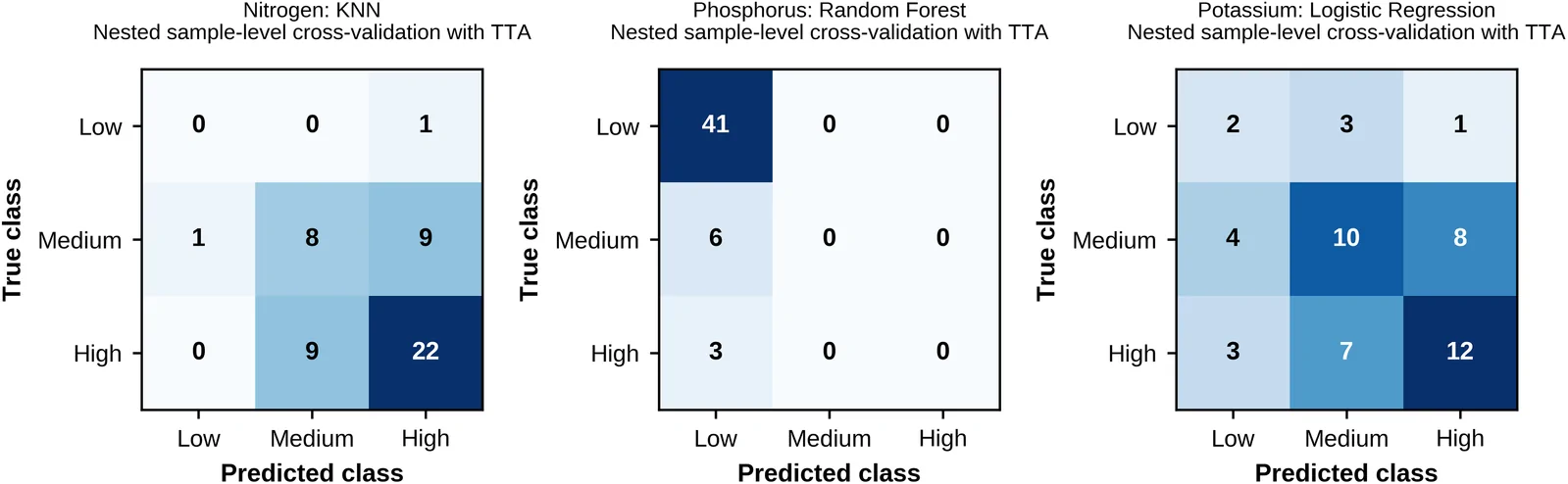
Confusion matrices of NPK for classical ML models.

**Fig 17:**
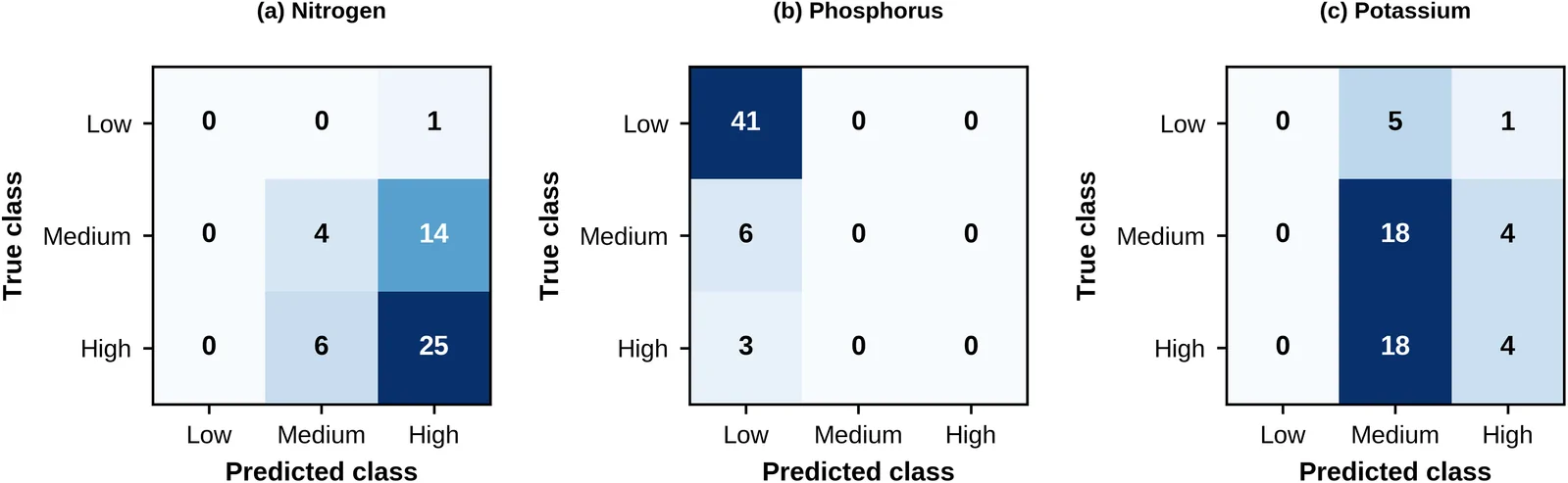
Confusion matrices of NPK for CNN models.

The matrices in both figures are in the order N, P, K. The ML models were better at recognizing the high-nitrogen and low-phosphorus samples, and the potassium model was able to make accurate predictions in all three classes. CNN also identified useful patterns, but the performance was not as high as the classical ML models.

## Discussion

The present study was conducted to show the feasibility of using controlled soil-extract images for the classification of nitrogen, phosphorus, and potassium status. One of the great features of the proposed framework is that it combines traditional nutrient lab analysis with standardization of image acquisition techniques, along with machine learning and deep learning methods. The controlled illumination system minimized unwanted variation, background, and environmental lighting effects on the images and helped the models to concentrate mainly on the color characteristics and images from the prepared soil extracts. The classical machine-learning models had an accuracy of 60%, 82%, and 48% for nitrogen, phosphorus, and potassium, respectively, and the compact CNN had the corresponding accuracy of 58%, 82%, and 44%, respectively. The results suggest that images of the soil may contain visual information that can be used to measure nutrient status; however, with the classical models, the nutrient discrimination overall was modest, and the phosphorus accuracy matched the majority-class baseline. The CNN results also suggest that automatic feature learning is possible, but the performance is still limited to the small and imbalanced dataset. One methodological contribution of this study is the use of sample-level validation, training-only augmentation, the use of majority-class baselines, balanced accuracy, macro-F1 score, Cohen’s kappa, and the use of confidence intervals, which provide a more transparent and reliable assessment than accuracy alone. Furthermore, the results from the present study must be treated with caution when compared with those of other studies that have reported higher accuracy, as they have generally tested different types of soil inputs such as raw-soil images, spectral measurements, sensors, or much larger data sets, but the current study focuses on chemically prepared soil extracts under controlled imaging conditions. Therefore, this study offers a unique and structured image-assisted NPK assessment. In order to evaluate external validity, further studies with larger and more balanced data sets, different soil types, geographical regions, imaging devices, and environmental conditions are required before any application based on a portable device or a smartphone could be considered. The controlled imaging setup was not an independently evaluated model component, but it was used to standardize image acquisition because matched images of samples under uncontrolled illumination or under other imaging conditions were not available; the isolated contribution of the controlled setup could not be quantified and should be investigated in future studies.

## Conclusion

This study developed a controlled image-based framework to classify the soil nitrogen, phosphorus, and potassium levels using chemically prepared soil extracts. Unlike the traditional image-based methods, which often rely on the raw visual surfaces of the soil, plant symptoms, remote sensing, or electronic sensor measurements, the proposed method analyzed the visual color and texture features resulting from the chemical extraction process. This strategy enabled laboratory-derived nutrient information to be connected with digital image features under standardized lighting conditions.

The best (evaluated) classical machine-learning models were KNN with an accuracy of 60% for nitrogen, Random Forest with an accuracy of 82% for phosphorus, and Logistic Regression with an accuracy of 48% for potassium. The nitrogen model was able to recognize the High category with good accuracy, and the phosphorus model was able to recognize all of the samples in the dominant Low category with good accuracy. The selected model for potassium was able to make predictions for all three nutrient classes, with performance better than the majority-class baseline. The Classical machine-learning models slightly outperformed the compact CNN for nitrogen and potassium, while both achieved the same accuracy for phosphorus, showing that well-designed color and texture information was more informative than deep features for the small number of samples used in the study. The overall findings suggest that there are visual patterns within the chemically extracted soil images that can be measured. In this way, this study establishes a structured foundation for combining chemical soil analysis, controlled imaging, and machine learning for NPK classification. This proposed soil nutrient assessment framework may offer a step towards more accessible, rapid, and image-assisted soil nutrient assessment while maintaining the scientific relationship between the chemical extractions and soil nutrient concentration.

In the future, the proposed framework should be evaluated on a larger and more representative dataset of soils, locations, depths, and seasons. External validation should also be performed to determine the generalizability of the models. Additional enhancements include color calibration, standardized illumination, improved image features, ensemble learning, and class-imbalance treatment. The framework can be expanded to a portable or smartphone-based method that enables quick NPK assessment with sufficient validation.

## Supporting information

S1 Checklist Completed PLOS questionnaire on inclusivity in global research. (DOCX)
