# Supplementary material for: Controlled image-based prediction of soil nutrient concentrations (NPK) using machine and deep learning
Source: PLoS One. 2026 Oct 27;21(10):e0360517. doi: 10.1371/journal.pone.0360517 (PMC13712755; doi:10.1371/journal.pone.0360517)
Supplement: Supplementary file 1 [file pone.0360517.s001.docx]

Inclusivity in global research

PLOS’ policy on inclusivity in global research aims to improve transparency in the reporting of research performed outside of researchers’ own country or community and ensures that PLOS publications reporting global research adhere to high standards for research ethics and authorship. Authors of relevant research articles may be asked to complete the questionnaire below, which outlines ethical, cultural, and scientific considerations specific to inclusivity in global research. This questionnaire may be requested when researchers have travelled to a different country to conduct research, if research uses samples collected in another country, research with Indigenous populations or their lands, or if research is on cultural artefacts. Researchers travelling to another country solely to use laboratory equipment will not normally be required to complete the questionnaire. However, the questionnaire can be requested at the journal’s discretion for any submission – if you have been requested to complete this questionnaire by the PLOS journal you submitted to, please do so.

Please complete the questionnaire below and include this as a Supporting Information file with your manuscript. Note that if your paper is accepted for publication, this checklist will be published with your article in the supporting information files. Please ensure that you reference the checklist in the main body of your manuscript. We suggest adding a subsection ‘Inclusivity in global research’ to your Methods section and adding the following sentence: “Additional information regarding the ethical, cultural, and scientific considerations specific to inclusivity in global research is included in the Supporting Information (SX Checklist)”

The questions have been designed to be applicable to a wide range of study types, and there are subsections for both human subjects research and non-human subjects research. If any of the questions are not relevant to your research please mark them as “N/A” as appropriate.

**Manuscript ID: PONE-D-26-13720**
*Controlled Image-Based Prediction of Soil Nutrient Concentrations (NPK) Using Machine and Deep Learning*
Completed for non-human environmental soil-sample research

**Ethical considerations, permits and authorship**

*This section is applicable to all research types.*

Provide details as to who granted permissions and/or consent for the study to take place in the Methods section of your manuscript. This should include the names of **all** ethics boards, governmental organizations, community leaders or other bodies that provided approval for the study. If individuals provided approval refer to these people by their role or title but do not list their name(s).

**Authors’ response:** The study involved the collection of soil samples from privately managed agricultural fields in Punjab, Pakistan. Access was granted by the respective field owners or field managers. Only small quantities of agricultural soil were collected, and the work did not involve protected areas, endangered species, human participants, Indigenous lands, archaeological sites, or culturally sensitive materials. Therefore, no formal governmental or institutional field-collection permit was required. This information is reported in the Methods subsection “Field-Site Access and Permits”; the final page number will follow the pagination of the submitted manuscript.

If there were any deviations from the study protocol after approval was obtained please provide details of these changes in the Methods section of your manuscript.
Did this study involve local collaborators that are residents of the country where the research was conducted or members of the community studied? If you do not have any authors from said communities, please provide an explanation for this below.

**Authors’ response:** No deviations from the stated soil-sampling, laboratory extraction, controlled imaging, or computational analysis procedures occurred. The study involved local collaborators and authors affiliated with institutions in Pakistan. Local faculty members provided guidance on appropriate soil-sampling procedures, and local laboratory staff assisted with soil-extract preparation and related experimental procedures. All individuals meeting PLOS authorship criteria are included in the author byline; technical assistance is acknowledged separately in the manuscript.

Everyone listed as an author should meet PLOS’ criteria for authorship and all individuals who meet these criteria should be included in the author byline, rather than the acknowledgements. For further information please see the journal’s Authorship Policy.

**Human subjects research (e.g. health research, medical research, cross-cultural psychology)**

Did you obtain written informed consent from a representative of the local community or region before the research took place? How did you establish who speaks for the community? Details of written informed consent obtained from study participants should be reported separately in the Methods section of your manuscript.

**Authors’ response:** N/A. The study did not involve human participants, interviews, surveys, medical data, or community representatives acting on behalf of human participants.

How did members of the local community provide input on the aims of the research investigation, its methodology, and its anticipated outcome(s)?

**Authors’ response:** N/A for human-subject research. Local faculty members contributed scientific guidance on appropriate agricultural soil-sampling methods.

When engaging with the local community, how did you ensure that the informed consent documents and other materials could be understood by local stakeholders?

**Authors’ response:** N/A. No informed-consent documents or other human-participant materials were used because the study did not involve human subjects.

Will the findings of the research be made available in an understandable format to stakeholders in the community where the study was conducted (e.g. via a presentation, summary report, copies of publications, etc.)? Please provide details of how this will be achieved.

**Authors’ response:** N/A for human-subject research. Nevertheless, the research article and the underlying sample-level dataset, original soil-extract images, documented classical machine-learning code, and README file are being made publicly available through PLOS ONE and Zenodo.

**Non-human subjects research using specimens/ animals collected as part of the study, or those housed in archival collections. Examples include archaeology, paleontology, botany and zoology.**

Did the permission you obtained from a local authority to perform the study include an agreement on access to outputs and benefit sharing? This may include procedures to enable fair distribution of the benefits and resources arising from the research performed. Please include any details of Prior Informed Consent and Benefit Sharing Agreements obtained. These may be required by field-specific regulations, for example the Convention on Biological Diversity (CBD) and the associated Nagoya Protocol.

**Authors’ response:** N/A. Routine agricultural soil samples were collected with permission from field owners or managers. The study did not involve protected biological or genetic resources, animals, endangered species, or materials governed by the Convention on Biological Diversity or the Nagoya Protocol. No formal Prior Informed Consent or Benefit Sharing Agreement was therefore required. The research outputs are being made publicly accessible through the article and the associated Zenodo record.

If the material used in your study was imported, please A) provide the year it was imported and B) indicate whether permits were obtained to import/export the materials used, C) provide details of any permits obtained. If this information is not available, please indicate this.

**Authors’ response:** N/A. The soil samples were collected and analysed within Pakistan. No study materials were imported or exported, and no import or export permits were required.

If you used archival specimens, please state how the material used in your study was acquired by the institute it is held in and provide details of any permits obtained for the original excavations/ sample collection. If this information is not available, please indicate this.

**Authors’ response:** N/A. No archival specimens, museum collections, excavated materials, or previously stored biological specimens were used in this study.

How was the potential cultural significance of the materials collected in your study to local communities considered in your research design? Were Indigenous peoples and/or local researchers and institutions involved with archaeological excavations / collection of specimens? If so, please provide a description of their involvement.

**Authors’ response:** The study involved routine agricultural soil samples from cultivated fields and did not include archaeological materials, human remains, Indigenous lands, sacred sites, or specimens known to have cultural significance. Local researchers and faculty members provided guidance on appropriate agricultural soil-sampling procedures, and access to the fields was granted by the respective owners or managers.

If your manuscript includes photographs of human remains please indicate whether authors obtained permission from descendants or affiliated cultural communities to do so.

**Authors’ response:** N/A. The manuscript does not include photographs of human remains, and the study did not involve human remains.
